# Towards super-clean graphene

**DOI:** 10.1038/s41467-019-09565-4

**Published:** 2019-04-23

**Authors:** Li Lin, Jincan Zhang, Haisheng Su, Jiayu Li, Luzhao Sun, Zihao Wang, Fan Xu, Chang Liu, Sergei Lopatin, Yihan Zhu, Kaicheng Jia, Shulin Chen, Dingran Rui, Jingyu Sun, Ruiwen Xue, Peng Gao, Ning Kang, Yu Han, H. Q. Xu, Yang Cao, K. S. Novoselov, Zhongqun Tian, Bin Ren, Hailin Peng, Zhongfan Liu

**Affiliations:** 1 Center for Nanochemistry, Beijing Science and Engineering Center for Nanocarbons, Beijing National Laboratory for Molecular Sciences, College of Chemistry and Molecular Engineering, Peking University, Beijing, 100871 P. R. China; 2 Academy for Advanced Interdisciplinary Studies, Peking University, Beijing, 100871 China; 3 Collaborative Innovation Center of Chemistry for Energy Materials (iChEM), State Key Laboratory of Physical Chemistry of Solid Surfaces, and The MOE Key Laboratory of Spectrochemical Analysis and Instrumentation, Department of Chemistry, College of Chemistry and Chemical Engineering, Xiamen University, 361005 Xiamen, China; 4 Beijing Key Laboratory of Quantum Devices, Key Laboratory for the Physics and Chemistry of Nanodevices, and Department of Electronics, Peking University, Beijing, 100871 P. R. China; 5 China Fortune Land Development Industrial Investment Co., Ltd Beijing, Beijing, China; 6 School of Physics and Astronomy, University of Manchester, Manchester, M13 9PL UK; 7 College of Chemistry and Chemical Engineering, Xiamen University, Xiamen, 361005 China; 8 Imaging and Characterization Core Lab, King Abdullah University of Science and Technology, Thuwal, 23955-6900 Saudi Arabia; 9 Physical Science and Engineering Division, King Abdullah University of Science and Technology, Thuwal, 23955-6900 Saudi Arabia; 10 Electron Microscopy Laboratory, and International Center for Quantum Materials, School of Physics, Peking University, Beijing, 100871 P. R. China; 11 Soochow Institute for Energy and Materials InnovationS (SIEMIS), College of Physics, Optoelectronics and Energy, Soochow University, Suzhou, 215006 China; 12 Jiangsu Provincial Key Laboratory for Advanced Carbon Materials and Wearable Energy Technologies, Soochow University, Suzhou, 215006 China; 13 Department of Chemical and Biomolecular Engineering Hong Kong University of Science and Technology Clear Water Bay, Hong Kong SAR, 999077 China; 14 Collaborative Innovation Center of Quantum Matter, Beijing, 100871 China; 15 Beijing Graphene Institute, Beijing, 100095 P. R. China

**Keywords:** Synthesis of graphene, Synthesis of graphene

## Abstract

Impurities produced during the synthesis process of a material pose detrimental impacts upon the intrinsic properties and device performances of the as-obtained product. This effect is especially pronounced in graphene, where surface contamination has long been a critical, unresolved issue, given graphene’s two-dimensionality. Here we report the origins of surface contamination of graphene, which is primarily rooted in chemical vapour deposition production at elevated temperatures, rather than during transfer and storage. In turn, we demonstrate a design of Cu substrate architecture towards the scalable production of super-clean graphene (>99% clean regions). The readily available, super-clean graphene sheets contribute to an enhancement in the optical transparency and thermal conductivity, an exceptionally lower-level of electrical contact resistance and intrinsically hydrophilic nature. This work not only opens up frontiers for graphene growth but also provides exciting opportunities for the utilization of as-obtained super-clean graphene films for advanced applications.

## Abstract (web summary)

The intrinsic properties of graphene and the resulting device performance are hindered by the impurities produced during the synthesis process. Here, the authors elucidate the origin of contaminations in CVD-grown graphene and devise a strategy towards the scalable production of ultra-clean graphene with >99% clean regions and low contact resistance.

## Introduction

Surface contamination has long been a great challenge in the whole society of carbon materials^1^, and still unresolved in graphene^2,3^. The surface contamination has been intensively highlighted as a major hurdle in probing intrinsic properties of graphene^3–6^, and strongly hinders device performance and applications of graphene, such as surface chemistry^7–10^ ultrahigh speed electronics^2^, and transmission electron microscopy (TEM) support^11^, where clean surface is highly needed. Among the various methods for graphene synthesis, chemical vapour deposition (CVD) approach, especially on Cu substrate, holds great potentials in the scalable and cost-efficient production in a controllable fashion^12^. Despite recent advances in dictating the grain size and scalability^13,14^ in CVD approaches, the growth of clean graphene films by eliminating surface contamination remains a daunting challenge^2^. Although much attention has been paid to post-growth processing aiming for higher degree of cleanness, with an emphasis on the suppression of transfer-related impurities^15–18^ and airborne contaminants^5^, the variations in reported cleanness indicate that the dominant processes and intrinsic root of contamination still remain to be unravelled^2,15^.

Herein, we prove that the contamination on graphene surface is primarily introduced during the high-temperature CVD growth. The growth of metre-scale, super-clean graphene with advanced performances is facilely realised through the continuous supply of Cu vapour, via an ingenious substrate design using alternating stacks of Cu foil and foam.

## Results

### Intrinsic contamination on graphene surface during growth

**Fig. 1:**
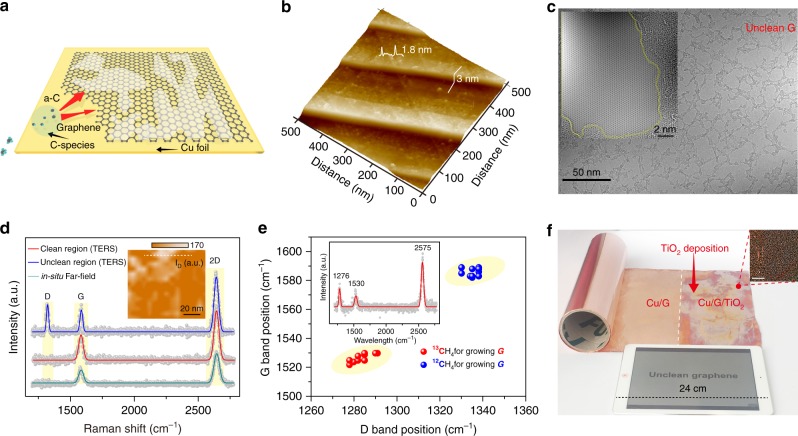
Unavoidable contamination on graphene surface during growth. **a** The competition between the formation of *sp*^2^-bonded graphene and defect-rich amorphous carbon (**a**–**c**) during the CVD growth of graphene. **b** AFM image of freshly prepared graphene on Cu foil after growth. **c** TEM image of a commonly as-grown graphene surface. Inset: HRTEM image of the clean and contaminated regions with atomic resolution. **d** TERS spectra of the unclean (blue line) and clean (red line) graphene regions in unclean graphene sample with Lorentzian line fit analysis, and in-situ far-field Raman spectrum of graphene in the same region (dark cyan line). Inset: TERS mapping of the D band intensity after smoothing. **e** Statistics of the D and G band positions from the contaminated regions of graphene grown by normal methane (blue) and ^13^C-labelled methane (red). Inset: representative TERS spectra of isotopically labelled graphene. **f** Photograph of 0.3 m × 1-m-sized unclean graphene after TiO_2_ visualisation. An iPad was served as a size reference. Inset: dark-field optical microscopy (OM) image of the graphene surface decorated by TiO_2_ particles. Note that, parameters such as the contact time and relative humidity of vaporised TiO_2_ particles were kept identical, in order for a better compassion of graphene cleanness. Scale bar: 100 μm

During the high-temperature catalytic growth of graphene on Cu, the graphene surface becomes simultaneously contaminated due to the generation of amorphous carbon, which has been widely reported to be stable in CVD conditions (Fig. 1a)^19^. The competition between the formation of *sp*^2^ crystalline carbon (graphitisation process) and amorphous carbon during a CVD reaction primarily determines the cleanness of the graphene surface, and this phenomenon has been discussed intensely in academic and industrial settings in relation to the preparation of synthetic graphite and diamond^19,20^. In the course of graphene growth, Cu would catalyse the decomposition of hydrocarbons as well as the graphitisation process. However, the catalytic activity of Cu would be limited gradually upon the increase of graphene coverage, presumably leading to the formation of amorphous carbon^19,21^.

Figure 1b shows an atomic force microscopy (AFM) view of graphene on the Cu substrate, right after growth. Sites of discontinuous surface contamination with a thickness of ~1 nm are clearly visible. The presence of surface contamination immediately after graphene growth seems quite ubiquitous, which was also observed in samples from other research groups (Supplementary Fig. 1). The high-resolution transmission electron microscopy (HRTEM) image of obtained graphene membrane transferred without the aid of polymer scaffold^15,22^ clearly manifests the universal distribution of amorphous carbon contaminants (Fig. 1c), leaving the clean graphene areas at only tens of nm^2^ (Fig. 1c, inset), in good agreement with previously reported observations^2,15^. Detailed elemental inspections further reveal that the contaminated regions are enriched with carbon and copper species (Supplementary Fig. 2). In addition, X-ray photoelectron spectroscopy (XPS) analysis identifies that the carbon species within the surface contamination contain *sp*^3^-carbon (Supplementary Fig. 3).

Tip-enhanced Raman spectroscopy (TERS) with improved lateral resolution and sensitivity^23^ was utilised to explore the composition and origin of the surface contamination. The representative TERS spectra of some graphene regions show prominent D (1350 cm^−1^) band signal (blue curve in Fig. 1d; Supplementary Fig. 4b), while the 2D-band mapping over the same area exhibited a high 2D band density, indicating the intrinsically high quality of the graphene (Supplementary Fig. 4c). The prominent D band in TERS was also observed in samples from other research groups (Supplementary Fig. 5). Therefore, the prominent D band intensity here is originated from the surface contamination rather than graphene itself, and was taken as the signature of amorphous carbon^24^. Note that, due to considerably higher spatial resolution and selective enhancement of D band intensity than that of conventional Raman techniques^23^, the observation of visible D band would be only possible in TERS (previous reports regarding TERS of graphene, see Supplementary Table 2), in contrast with in-situ far-filed result (dark cyan) and results in conventional Raman spectroscopy (Supplementary Fig. 6 and Supplementary Table 1). Furthermore, the TERS mapping using the D band (Fig. 1d, inset) shows a similar distribution to that of contamination obtained by AFM. In this way, through TERS, we can differentiate between the clean graphene regions (with nearly no D band, red curve in Fig. 1d) and contaminated graphene regions (high D band intensity, blue curve in Fig.1d).

To clarify the origin of the surface contamination, a ^12^C/^13^C isotope-labelling technique^21^ was employed (Fig. 1e). The evident difference in the contamination-related peaks between the ^12^C and ^13^C samples indicates that amorphous carbon has already been isotopically labelled by the carbon feedstock employed in the CVD procedure. In addition, time-of-flight ion mass spectrometry (ToF-SIMS) analysis verifies that the carbon source fuels the dual formation of graphene and amorphous carbon during CVD (Supplementary Fig. 7). Altogether, our results unambiguously reveal that surface contamination is mainly introduced during the high-temperature synthetic process.

The direct and large-area visualisation of amorphous carbon on graphene/Cu foil is the key to the exploration of contamination mechanism towards the growth of clean graphene. This can be readily achieved through the exposure of as-grown graphene/Cu to TiCl_4_ vapour in humid air, where TiO_2_ nanoparticles were in-situ deposited on the graphene/Cu^25^. Interestingly, these TiO_2_ nanoparticles would be preferentially and selectively adsorbed onto the contaminated regions of graphene (Supplementary Fig. 8). Thanks to the strong Mie-scattering ability of TiO_2_ nanoparticles to visible light, the as-grown, large-area graphene surface turns multi-coloured after TiO_2_ deposition, in sharp contrast to the unexposed graphene/Cu sheets (Fig. 1f), thereby indicating the adsorption of large amounts of TiO_2_ particles on a predominantly unclean surface (Fig. 1f, inset). This urges us to pay further attention to the advancement of CVD synthetic protocols leading to the production of super-clean graphene films at a large scale.

### Growth of metre-scale, super-clean graphene

**Fig. 2:**
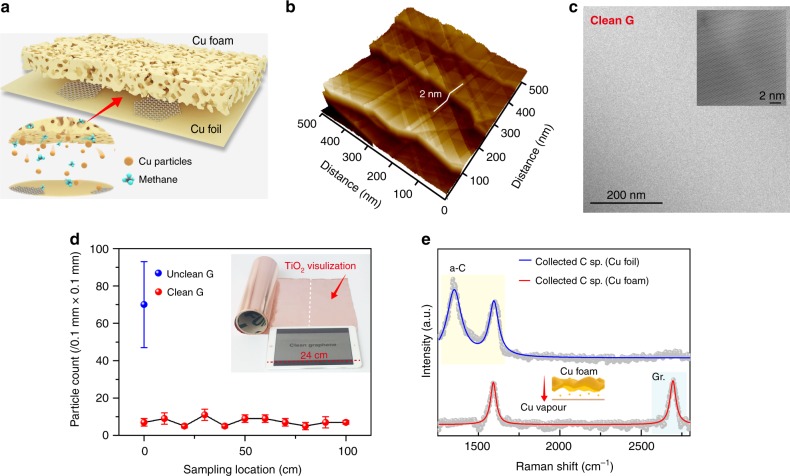
Growth of metre-scale, super-clean graphene. **a** Schematics of the experimental design, i.e., the Cu foil-foam stacked structure, for the growth of super-clean graphene. Inset: Illustration of interaction of Cu and methane in the gap between Cu foil and foam. **b** AFM image of the freshly prepared clean graphene on Cu foil after growth. **c** TEM image of the super-clean graphene membrane. Inset: HRTEM image of the graphene lattice. **d** Number of TiO_2_ particles adsorbed on super-clean graphene at different positions and on unclean graphene. The error bar represents the relative deviation. Inset: photograph of metre-scale, super-clean graphene after TiO_2_ visualisation. **e** Raman spectra of the carbon species (C sp.) on Cu nanoparticles collected in boundary layer during CVD growth, with (red line) and without (blue line) the assistance of Cu foam. The nanoparticles were collected on quartz substrates (see details in supporting information). The yellow and blue rectangles represent amorphous carbon and graphene-related Raman peaks, respectively

Based on the aforementioned insights, we devise a substrate architecture via stacks of Cu foil and Cu foam (Fig. 2a) (Cu foil-to-foam distance of ~15 μm, Supplementary Fig. 9) to realise the controlled fabrication of super-clean graphene films. The key aspect is an ingenious catalyst design utilising Cu foam mediation leading to the growth of super-clean graphene without detectable contaminations, as witnessed by AFM (Fig. 2b). The as-obtained graphene was further characterised by HRTEM (Fig. 2c), showing continuous, clean sheets with negligible amorphous carbon regions and easily observable atomic lattices (Fig. 2c, inset). Indeed, our prepared graphene possesses much higher degree of cleanness (accounting for >99% clean regions) as compared with the previously reported data and methods (Supplementary Table 3 and Supplementary Fig. 10)^15,26–28^. This confirms the feasibility and significance of our protocol design in obtaining super-clean graphene, with emphasis on suppression of contamination during growth stage. In addition, the quantitative measurement of cleanness of graphene sample is presented in Supplementary Fig. 11.

Folding the copper foil to form an alternating foil/foam stacked roll leads to the growth of large-scale (0.3 m × 1 m), super-clean graphene (Supplementary Fig. 12). Upon exposure to the vaporised TiO_2_, the graphene/Cu shows no colour change to the naked eyes (Fig. 2d), suggesting a significantly lower-level adsorption of TiO_2_ nanoparticles and hence a clean surface of grown graphene as compared with the contaminated films prepared via traditional route (Supplementary Fig. 8b, c). Notably, the design of stacked architecture, rather than carbon precursor and/or surface morphology of the substrate, stays as the key parameter in determining the realisation of super-clean graphene. In this sense, sub-centimetre-sized, single-crystalline graphene with super-clean surface can be achieved by using controllably limited carbon supply (Supplementary Fig. 13)^29^.

During a CVD reaction, Cu substrate would catalyse the decomposition of carbon feedstock and graphitisation process, whose catalytic ability, in turn, would determine crystalline quality of carbon material, i.e., the generation of crystalline graphene or amorphous carbon. In such case, gradually inhibited catalytic ability of Cu due to the increasing coverage of graphene enables the formation of amorphous carbon onto the graphene sheet during the growth (Supplementary Fig. 14a, b). Produced by the decomposition of carbon precursor, a large quantity of carbon species would desorb from Cu substrate, being abundant in the boundary layer to induce the generation of amorphous carbon to contaminate graphene (Fig. 2e; Supplementary Fig. 14c–f and Supplementary Fig. 15)^30,31^. Hence, the catalytic activity of Cu is relatively insufficient in a conventional CVD reaction. In contrast, due to its high specific surface area, the use of Cu foam mediation is expected to provide sufficient supply of Cu vapour, to unintermittently catalyse the decomposition of carbon species. Then, the formation of amorphous carbon would be suppressed, enabling the growth of crystalline graphene with super-clean nature (Fig. 2e; Supplementary Fig. 14g, i). Note that at low pressure, the Cu foil itself can provide Cu vapour, such as previous reported Cu envelop structure^32,33^, which can be also used for the remote catalytic growth of graphene on oxide substrates^34^. However, the amount of Cu vapour is very limited, and especially it would be reduced with the increasing coverage of graphene on Cu foil. Thus, a continuous supply of sufficient Cu vapour is very important for suppressing the formation of amorphous carbon. In this regard, the lower growth rate of graphene on Cu foam than that on Cu foil guarantees a continuous supply of Cu vapour during the entire growth process, which is the key factor in obtaining clean graphene (Supplementary Fig. 16).

### Super-clean graphene surface after transfer onto target substrates

**Fig. 3:**
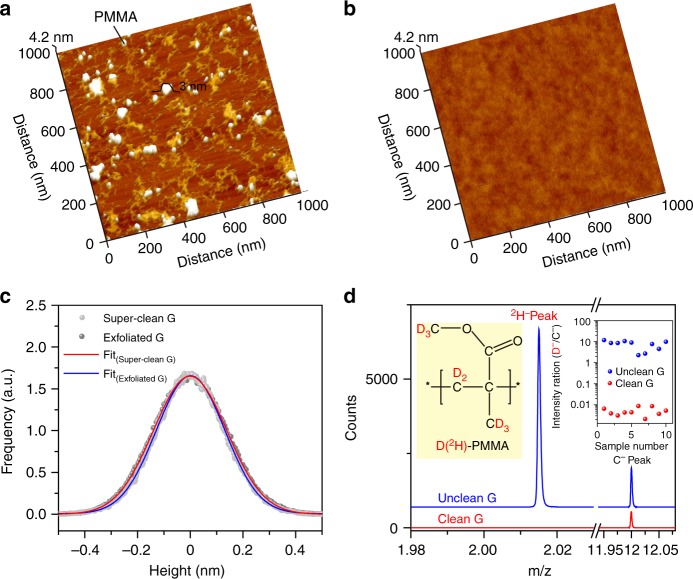
Super-clean graphene surface after transfer onto target substrates. **a**–**b** AFM images of transferred super-clean graphene (**a**) and unclean graphene (**b**) onto SiO_2_/Si substrates. **c** Height histograms for super-clean graphene (grey, red line) and exfoliated graphene (black, blue line) on SiO_2_ substrate. The data of super-clean graphene is obtained from AFM image (**b**). **d** ToF-SIMS spectra of transferred super-clean (red line) and contaminated graphene (blue line) on SiO_2_/Si substrate after removal of ^2^H-PMMA. Note that the transfer process is kept the same for super-clean and unclean graphene. Inset (left): structural formula of ^2^H-PMMA. Inset (right): statistics of ^2^H^-^ peak intensity of super-clean (red) and contaminated graphene (blue), as obtained from ToF-SIMS results

Transferring graphene onto target substrates, such as SiO_2_/Si substrate, is needed for further device fabrication and applications. However, after transfer, the common CVD-grown graphene suffers from an abundant distribution of polymer residues, such as polymethyl-methacrylate (PMMA) residues (Fig. 3a). Interestingly, the availability of super-clean graphene surface on Cu foil ensures a clear reduction of polymer impurities after transfer, as confirmed in the AFM image, where polymer residue and the amorphous carbon are clearly invisible (Fig. 3b). The surface cleanness is comparable with mechanically exfoliated counterpart with only substrate-induced small fluctuation, evident from the similar height histogram (Fig. 3c). In addition, this small fluctuation becomes invisible after transferring super-clean graphene onto atomically flat mica substrate (Supplementary Fig. 17). For a large-area evaluation of cleanness, graphene was transferred with the assistance of deuterated PMMA (^2^H-PMMA). Therefore, it was possible to quantify the amount of PMMA residues using mass signature of isotope ^2^H (Fig. 3d, inset). After the removal of ^2^H-PMMA by acetone, ToF-SIMS was conducted to detect the amount of the ^2^H on graphene surface. For unclean graphene sample after transfer, a clear ^2^H^−^ peak is observed at the m/Z of 2, indicating the abundant PMMA residues. In contrast, ^2^H^−^ peak is invisible in clean sample, confirming clear reduction of transfer-related impurities on graphene surface after the transfer (Fig. 3d). The large-scale cleanness is additionally confirmed by the statistic of intensities of ^2^H^−^ peaks of unclean and super-clean graphene surface after transfer (Fig. 3d, inset).

### Optical and electrical properties of super-clean graphene

**Fig. 4:**
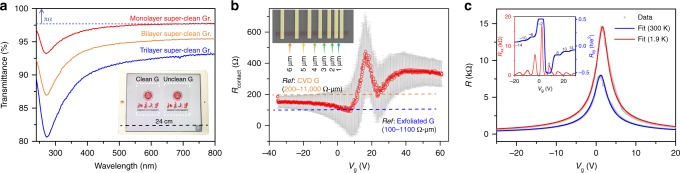
Optical and electrical properties of super-clean graphene. **a** UV-vis spectra of monolayer (red line), bilayer (orange line) and trilayer (blue line) super-clean graphene films on quartz substrates. Inset: a photograph of large-area, super-clean graphene and unclean graphene transferred onto PET substrates. An iPad served as a size reference. Note that multilayer graphene was fabricated by layer-by-layer transfer. The contrast is enhanced for clarity. A slight fluctuation of measured values is visible across the entire measured spectrum, which might result in a small overshoot. **b** Measured contact resistance as a function of gate voltage. The orange and blue dashed lines denote the reported contact resistance of the CVD-grown and mechanically exfoliated graphene, respectively. Inset: false-coloured scanning electron microscopy (SEM) image of the measured devices. The channel lengths vary from 1 to 6 μm. The error bar represents the relative deviation. **c** Typical plot of the resistance of graphene as a function of the gate voltage (*V*_g_) at room temperature (blue line) and 1.9 K (red line). Inset: longitudinal (*R*_*xx*_, red line) and Hall resistance (*R*_*xy*_, blue line) of super-clean graphene at magnetic field intensities of 5 T at 1.9 K. Contact metal: Pd/Au, 10/80 nm. The fitting is based on nonlinear fitting method (see Supplementary Fig. 22)

Our super-clean graphene, almost devoid of amorphous carbon (>99.0% clean regions), represents the quality of CVD-derived graphene with optical and electrical properties comparable with that obtained by mechanical exfoliation. The large-area transfer of clean and unclean samples onto transparent polyethylene terephthalate (PET) substrates was achieved by means of a thermal release tape method^14^, where super-clean graphene sample exhibits a lighter contrast than that of the unclean counterpart (Fig. 4a, inset). The elimination of amorphous carbon and low-level of polymer residues after the transfer altogether contribute to the enhanced light transparency in both monolayer and multilayer graphene films (Fig. 4a; Supplementary Fig. 18).

From the point of view of device applications, the construction of high-performance graphene transistors relies heavily upon the reliable fabrication of electrical contact and graphene channel with high carrier mobility^35^. In our study, we found that commonly used contact metals (Au, Cr and Pd) were preferentially adsorbed on the contaminated regions upon deposition (Supplementary Fig. 19). This would result in small grain sizes of the deposited metal and less effective contact between the metal and graphene. Furthermore, transfer-related polymer residues would lead to a larger coupling length and reduce the coupling strength between the metal and graphene, giving rise to larger contact resistances^36^. In contrast, using a graphene transistor array (six transistors for transfer length method (TLM) measurements) (Fig. 4b, inset)^37^, the extracted contact resistance of super-clean graphene devices is 115 ± 19 Ω μm with minimum value of 96 Ω-μm at room temperature when the channel is slightly p-doped (Fig. 4b; Supplementary Fig. 20), substantially lower than the previously reported values (Supplementary Table 4) and that of unclean graphene with similar work function, confirming the importance of improving cleanness for better contact^36,37^ (Supplementary Fig. 21).

Furthermore, the measured field-effect transistor mobility of the transferred graphene on SiO_2_/Si substrate ranges from 14,900 to 18,500 cm^2 ^V^-1^ s^-1^ at room temperature and ~31,000 cm^2^·V^-1^·s^-1^ at 1.9 K (Fig. 4c), higher than those of the unclean samples and previously reported values of CVD graphene on SiO_2_ substrates (Supplementary Fig. 22, Supplementary Tables 5, 6). Besides the suppression of amorphous carbon, the reduction of polymer residue was reported to result in an enhanced carrier mobility^4^. The carrier mobility of graphene is also sensitive to the transfer process and water doping. Thus, the transfer process is carefully controlled to obtain a convincing comparison (see the Methods section). The exceptional quality of obtained graphene was further corroborated by the evolution of the quantum Hall effect with magnetic field in devices, where the prominent quantum Hall platform is visible at low magnetic field intensities (Fig. 4c, inset).

Encapsulation of graphene would significantly improve the carrier mobility due to the reduced transfer-related doping, substrate scattering and squeezing effect of *h*-BN to enhance the interface cleanness^38–40^. Following reported encapsulation techniques^38,39^, in encapsulated as-grown clean graphene samples, the carrier mobility can be further improved to 625,000 cm^2^ V^-1^ s^-1^ for hole side and 1,083,000 cm^2^ V^-1^ s^-1^ for electrons side, along with observation of ballistic transport at 1.9 K (Supplementary Fig. 23). The reduced and uniform full-width at half maximum (FWHM) of 2D in Raman results were also observed, as an indicator of enhanced carrier mobility in encapsulated graphene (Supplementary Fig. 24)^41^. Consequently, the improved cleanness and optimisation of transfer techniques, such as significant improvement in the fabrication of large and thin *h*-BN flakes would be both important for future electrical application of graphene^38^.

Furthermore, super-clean graphene also exhibits an intrinsically hydrophilic property (Supplementary Fig. 25) and higher thermal conductivity (Supplementary Fig. 26) and electrical conductivity (Supplementary Fig. 27) than that of unclean graphene.

## Discussion

In essence, our results elucidate the origins of surface contamination of CVD graphene and outline a possible path towards the large-scale production of super-clean graphene with reliable high-quality equivalent to that of the mechanically exfoliated counterpart by designing a Cu foam mediator. The method presented here not only opens up avenue for advanced applications of graphene at the industrial level but also promotes further studies on the tailored synthesis of other two-dimensional crystals with super-clean nature.

## Methods

### Growth of super-clean graphene films

The super-clean graphene was grown by the low-pressure CVD method. The Cu foil (25 μm-thick, 99.8%, Alfa Aesar) after electrochemical polishing was placed below a Cu foam (840 g^1^·m^−2^ in areal density and 1.0 -mm thickness) (Supplementary Fig. 9) and then they were loaded into CVD system equipped with a 2.5-cm-diameter quartz tube. The system was heated to 1030 °C in 1 h with H_2_ (200 sccm, ~102 Pa), followed by annealing in H_2_ (200 sccm, ~102 Pa) for 1 h to eliminate the surface oxygen and contamination. Subsequently, CH_4_ was introduced to initiate the graphene growth for different duration. Note that, the molar ratio of CH_4_ and H_2_ was carefully controlled to produce large graphene single crystals. The flow rate of CH_4_ is ~0.1–1.0 sccm with pressure from 0.3 Pa to 2.8 Pa. After growth, the system was quickly cooled down to room temperature while still under the same flow. The ^13^C-isotope-labelled methane is purchased from the Sigma-Aldrich company (production number #490229) with ^13^C atom ratio of 99%. The ^2^H-PMMA is purchased from Polymer source company (production number #P100226-dsPMMA) with M_n_ = 820,000 and M_w_ = 1,500,100.

The domain size of graphene single crystals grown using the vertical stacking structure of Cu foil and Cu foam can be tuned from tens of micrometres to large than three millimetres by carefully controlling the carbon source supply (H_2_:CH_4_ molar ratio). The domain size distribution of graphene grown using different H_2_:CH_4_ molar ratio is 22.5 ± 1.2 μm for H_2_:CH_4_ molar ratio of 200, 125 ± 14 μm for H_2_:CH_4_ molar ratio of 300, 350 ± 11 μm for H_2_:CH_4_ molar ratio of 400, 750 ± 80 μm for H_2_:CH_4_ molar ratio of 500, 1750 ± 152 μm for H_2_:CH_4_ molar ratio of 1000, 2500 ± 575 μm for H_2_:CH_4_ molar ratio of 2000, respectively.

### Graphene transfer

The graphene was transferred onto quartz or SiO_2_/Si substrate by the PMMA method. The graphene film was spin-coated with PMMA and baked at 170 ^o^C for 5 min. Then, 1 M Na_2_S_2_O_8_ solution was used to etch Cu foil away. After being washed by deionized water, PMMA/graphene was subsequently placed onto target substrates. Especially, for reducing transfer-related doping, a dry transfer method was used, where a window cut in scotch tape was applied to the backside of Cu before etching Cu substrate. After etching, the resulting suspended graphene/PMMA/membrane across the tape window can be rinsed by isopropanol and then dried overnight. Subsequently, the membrane was adhered onto a heated SiO_2_/Si substrates at 150 ^o^C. Finally, PMMA was dissolved by acetone. The super-clean graphene on Cu was transferred onto TEM grid without the assistance of PMMA to endure no interference from polymer residue. In detail, by the evaporation of isopropanol drop between graphene on Cu and TEM grid, a TEM grid is directly contacted with graphene as a supporting layer in the subsequent etching and drying process.

### TERS measurement

TERS measurements were performed on a modified upright TERS system (NTEGRA Spectra, NT-MDT) in scanning tunnelling microscopy (STM) mode. A long working distance objective (×100, NA 0.7) was used for both excitation and collection of the backscattered light from the sample. The exciting wavelength was 632.8 nm and the laser power on the sample was less than 0.5 mW to avoid sample degradation. All the TERS spectra were collected with an acquisition time of 1 s.

### ToF-SIMS measurement

Annealing experiments were performed in the chamber of the ToF-SIMS spectrometer (ToF-SIMS V, ION-TOF GmbH, Munster, Germany) before characterisation. The samples were analysed at 25 °C and after annealing at 100 °C for 1 h. ToF-SIMS spectra were acquired at the annealing temperature using a Bi_3_^+^ beam operating at 25 keV. The scanning area was 200 μm × 200 μm with an acquisition time of 40 s. Negative ion spectra were collected for each sample. The software used for peak analysis was SurfaceLab 6.0 from ION-ToF.

### Graphene characterisation

Raman spectra were obtained with LabRAM HR-800 with 514nm laser and ×100 objective. Optical microscopy images were obtained with an Olympus BX51 microscopy. Optical transmittance spectra were collected by a Perkin-Elmer Lambda 950 UV-vis spectrophotometer.

SEM images were obtained with SEM (Hitachi S-4800, acceleration voltage 5–30 kV). The graphene on TEM grids was characterised by TEM (FEI Tecnai F30, acceleration voltage 300 kV). HRTEM imaging was performed on an aberration-corrected and monochromated G^2^ cubed Titan 60–300 electron microscope under 80 kV. Energy-dispersive X-ray (EDX) spectra, bright-field (BF) and high-angle annular dark-field (HAADF) images were acquired using scanning TEM (STEM) mode at 60 kV in a double corrected FEI Titan Themis G2 electron microscope.

The graphene samples were transferred onto SiO_2_/Si substrates with marks for alignments and then subjected to AFM (Veeco dimension 3100) imaging to determine whether they were flat without wrinkle. Next, each graphene sample was etched into a Hall bar geometry or graphene transistor array using a PMMA etching mask (PMMA 950 K A2 @ 4000 rpm) designed by electron-beam lithography (EBL) (Raith 150 2nd) and reactive-ion etching (RIE) with O_2_ (Trion Technology Minilock III). Finally, after using EBL to design a PMMA mask (PMMA 950 K A4 @ 4000 rpm), 10-nm Pd and 80-nm Au were deposited on the samples using an electron-beam evaporator (Kurte J. Lesker AXXIS) and then a standard metal lift-off technique. Electrical characterisation at room temperature was performed in a vacuum probe station (Lakeshore TTP-4) using a Keithly Semiconductor Characterisation System (Model 4200-SCS). Electrical-transport and magneto-transport measurements at low temperatures were performed using a lock-in amplifier (Stanford Research 830) at 17 Hz with a source current of 10–100 nA.

The element analysis was performed by XPS (Kratos Analytical AXIS-Ultra with monochromatic Al Kα X-ray). AFM characterisation of graphene on Cu was carried out on Bruker dimension icon microscopy using the Scanasystg mode. The water contact angles were measured on a Dataphysics OCA 20 contact-angle system at room temperature.

## Supplementary information

Supplementary Information

Peer Review File

## Data Availability

The authors declare that the data supporting this study are available within the article and its Supplementary Information files. Further information is also available from the corresponding authors upon reasonable request.
