# Supplementary material for: Towards super-clean graphene
Source: Nat Commun. 2019 Apr 23;10:1912. doi: 10.1038/s41467-019-09565-4 (PMC6478734; doi:10.1038/s41467-019-09565-4)
Supplement: Supplementary file 1 — Supplementary Information [file 41467_2019_9565_MOESM1_ESM.pdf]

Supplementary information for:

Towards super-clean graphene

Li Lin et al.

Supplementary Figure 1: AFM characterization of chemical vapor deposition (CVD)-grown graphene surfaces on Cu foil, obtained from other labs.

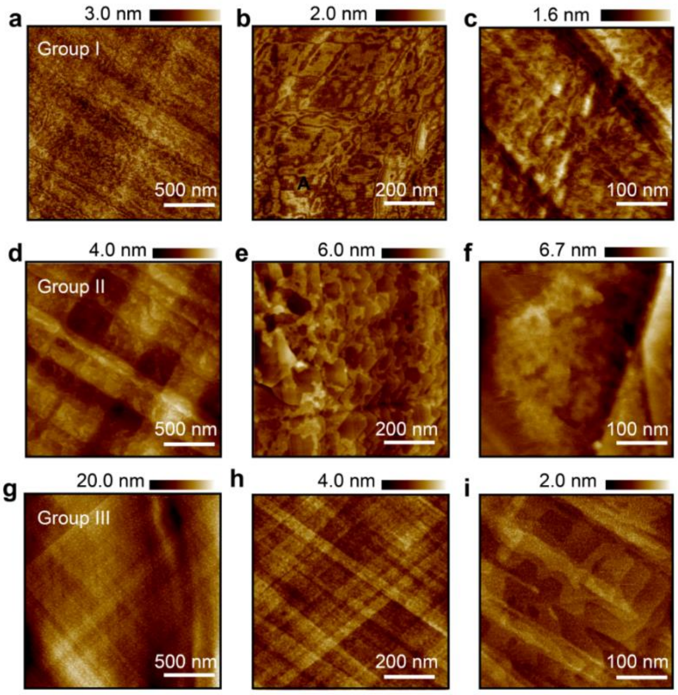

Supplementary Figure 1. AFM images of CVD-grown graphene surfaces on Cu foil obtained from other three laboratories. Group I (a-c), Group II (d-f), and Group III (g-i).

Supplementary Figure 2: Elemental composition of surface contamination on graphene surfaces.

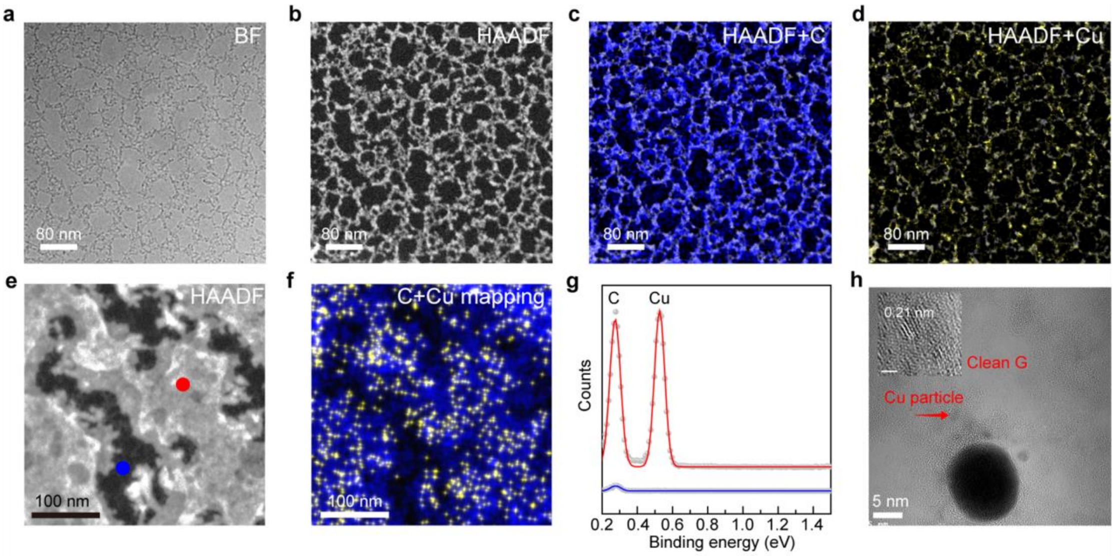

Supplementary Figure 2. Elemental composition of surface contamination on graphene surfaces. a-d, BF image (**a**), HAADF-STEM (**b**) and HAADF-STEM plus STEM-EDX of C (**c**) and Cu (**d**) maps of graphene surface, which is transferred onto TEM grid without the assistance of polymer. Note that the carbon distribution is consistent with that of amorphous carbon because the contaminated region exhibits a stronger carbon signal compared to that of the clean graphene region. **e**, **f**, HAADF-STEM (**e**) and HAADF-STEM plus scanning STEM-EDX C and Cu map (**f**) of graphene surface. **g**, EDX spectra of marked region in (**e**). The blue zone, corresponding to the clean region, displays a weak carbon signal, while the red zone, corresponding to the contaminated region, exhibits a stronger carbon signal, along with the strong Cu signal. **h**, BF TEM image of the graphene membrane with the presence of Cu nanoparticles in contaminated region. The inset shows HRTEM image of one particle. Scale bar: 1 nm.

Supplementary Figure 3: X-ray photoelectron spectroscopy (XPS) analysis of graphene on copper, immediately after growth

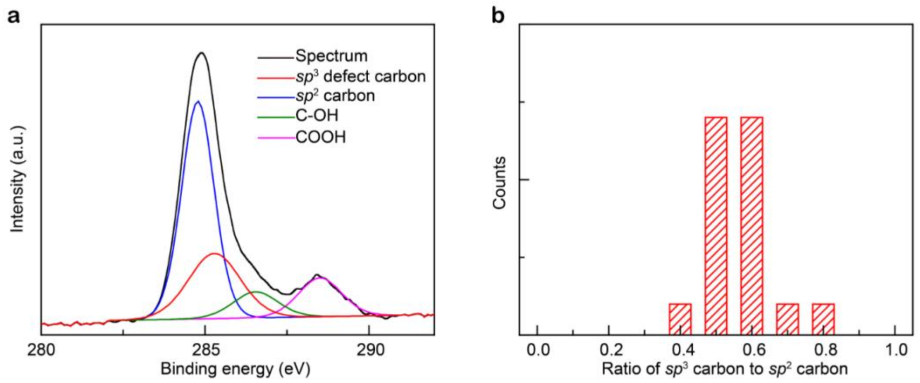

Supplementary Figure 3. XPS analysis of the graphene on copper. a, Representative C 1*s* spectrum of graphene on Cu foil, immediately after growth. **b**, Statistical ratios of s*p*^{2}-bonded carbon to s*p*^{3}-defective carbon in CVD-grown graphene samples, as calculated from the corresponding XPS data. For better comparison, some samples were obtained from other research groups; some were also grown according to the common methods.

Supplementary Figure 4: Tip-enhanced Raman spectra (TERS) of graphene surface.

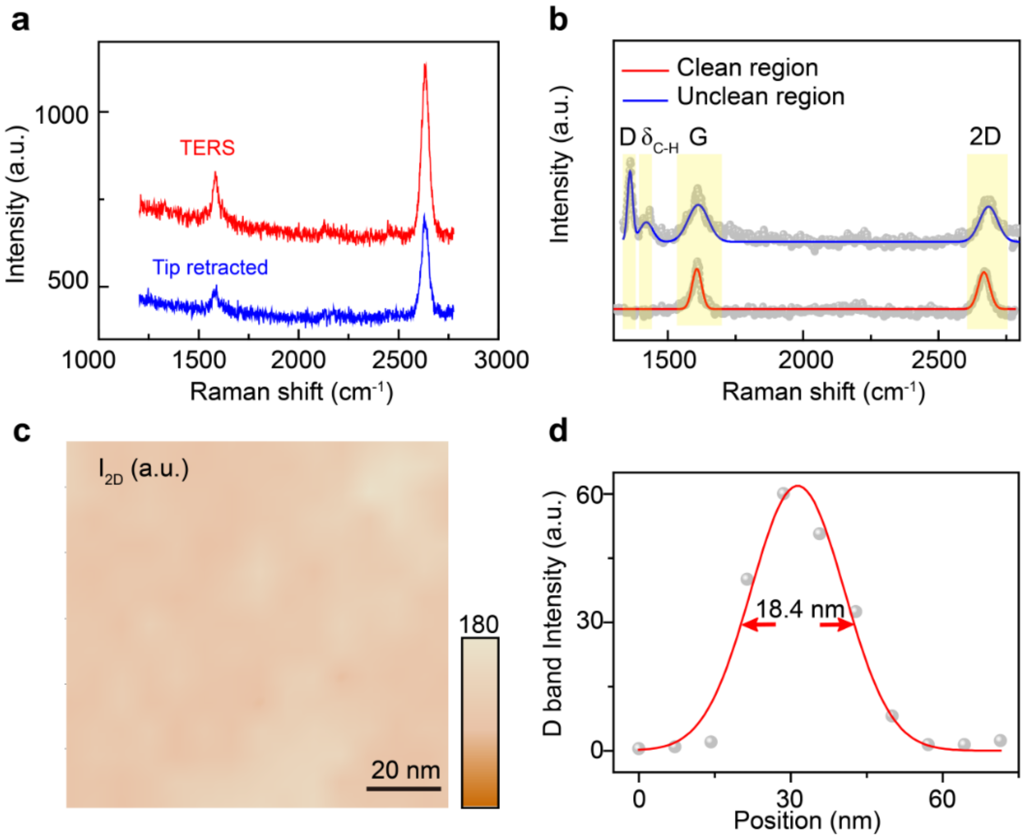

Supplementary Figure 4. a, TERS (red line) and far-field Raman signal (blue line). For both spectra, laser power is 0.25 mW and the acquisition time is 1 s. **b**, Typical TERS spectra of clean and unclean regions, indicating the presence of C-H bending peak. **c**, Corresponding 2D band intensity map of same region in inset of Fig. 1d. **d**, The D band intensity changes along one line in Fig. 2d.

Supplementary Figure 5: TERS analysis of samples from other labs.

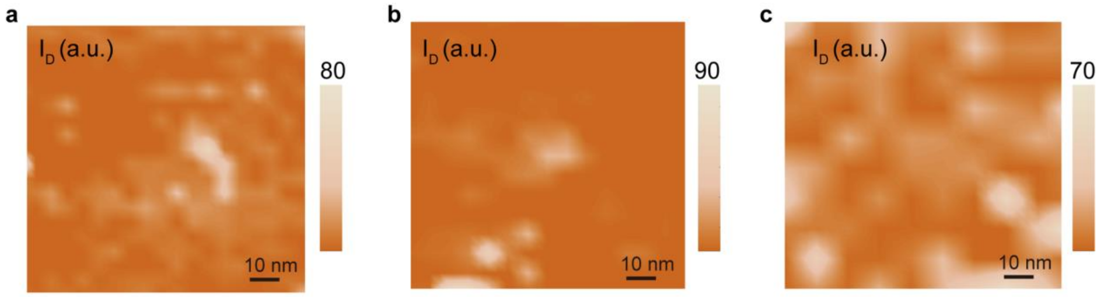

Supplementary Figure 5. TERS mapping of D band intensity of the graphene sample from three representative graphene research groups, as mentioned in supplementary Figure 1. Group I (a), Group II (b), and Group III (c).

Supplementary Figure 6: Confocal Raman analyses of graphene

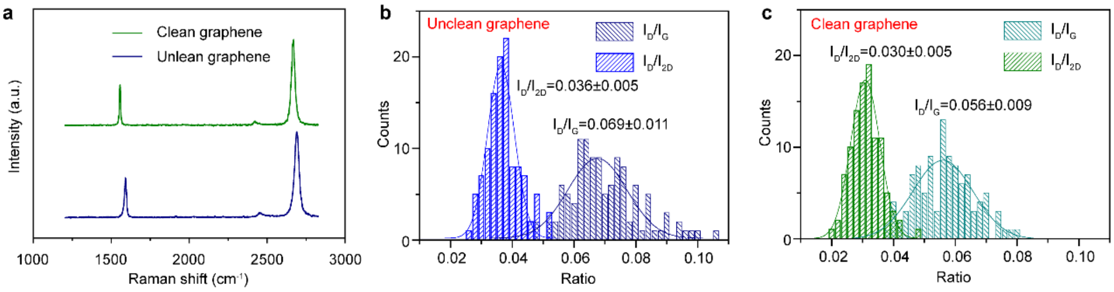

Supplementary Figure 6. Confocal Raman analyses of graphene. a, Confocal Raman analysis of clean graphene (green line) and unclean graphene (blue line), indicating no D band is observable in conventional Raman spectra regardless of the cleanness. **b-c**, Statistics of the I_D/I_G ratio and I_D/I_{2D} ratio, obtained from the conventional Raman spectra of the unclean graphene (**b**) and clean graphene sample (**c**).

Supplementary Figure 7: Time-of-flight ion mass spectrometry (ToF-SIMS) characterization of isotopically labeled graphene.

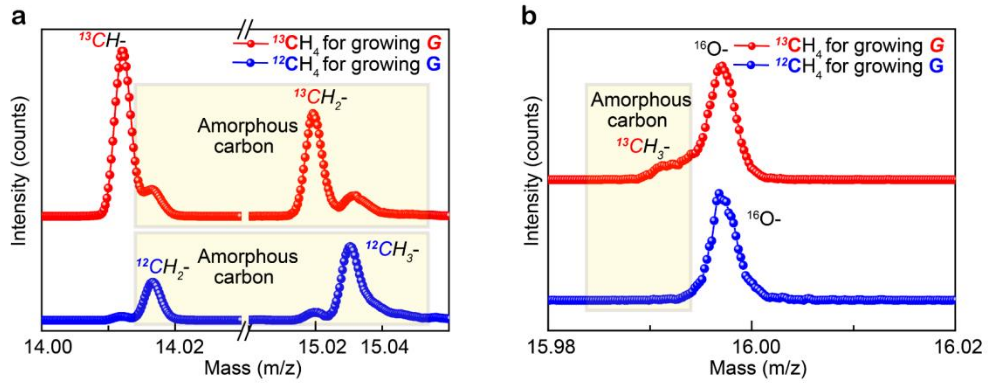

Supplementary Figure 7. ToF-SIMS spectra of ^{12}C-graphene (blue) and ^{13}C-graphene (red) covering the CH^- ion peak and CH_2^- ion peak (a), and CH_3^- ion peak (b). The yellow rectangles identify peaks related to amorphous carbon. Note that the peak of CH_2^- is amplified by a factor of 20 for a better demonstration that amorphous carbon is isotopically labeled.

Supplementary Figure 8: Visualization of amorphous carbon on graphene surfaces using TiO_2 particles.

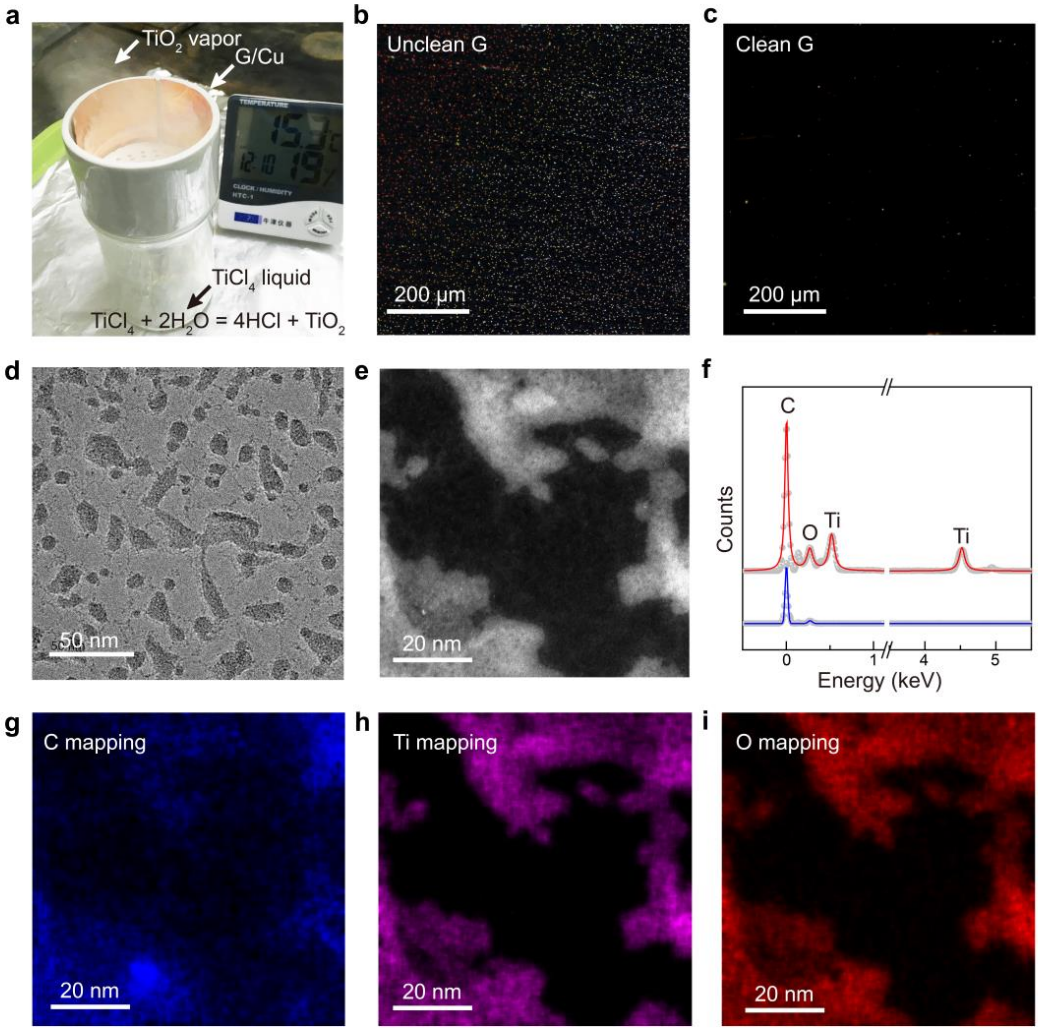

Supplementary Figure 8. Visualization of amorphous carbon on graphene surfaces using TiO_2 particles. **a**, Photograph of the experimental setup for the deposition of TiO_2 nanoparticles on graphene surfaces for visualizing amorphous carbon. **b**, **c**, Dark field (DF) optical microscopy (OM) image of the unclean (**b**) and clean (**c**) graphene surface on Cu foil after the TiO_2 visualization. **d**, Representative TEM image of a graphene surface after TiO_2 deposition. **e**, HADDF-STEM image of a graphene surface after TiO_2 deposition. **f**, Corresponding EDX spectra of the clean (green line) and contaminated (blue line) region marked in (**b**). **g-i**, STEM-EDX C (**g**), Ti (**h**) and O (**i**) maps of the region in (**e**).

Supplementary Figure 9: The structures of vertically stacked Cu foil and Cu foam.

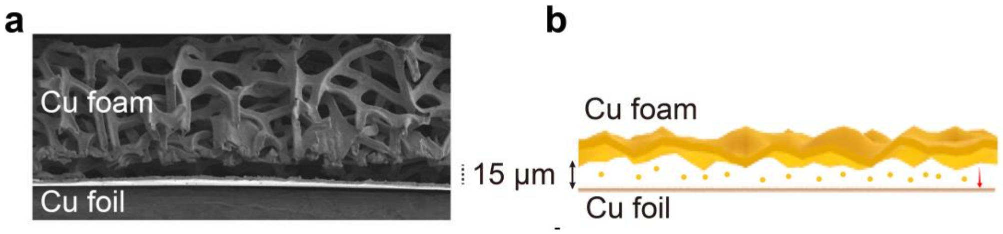

Supplementary Figure 9. The structures of vertically stacked Cu foil and Cu foam. a, Typical SEM cross-sectional image of the vertically stacked structure of Cu foil and foam. The gap is around 15 μm. **b**, Schematic of the vertically stacked structure depicted in (**a**) displaying an improved supply of Cu vapor in the small gap.

Supplementary Figure 10: The TEM characterization of graphene with different cleanness.

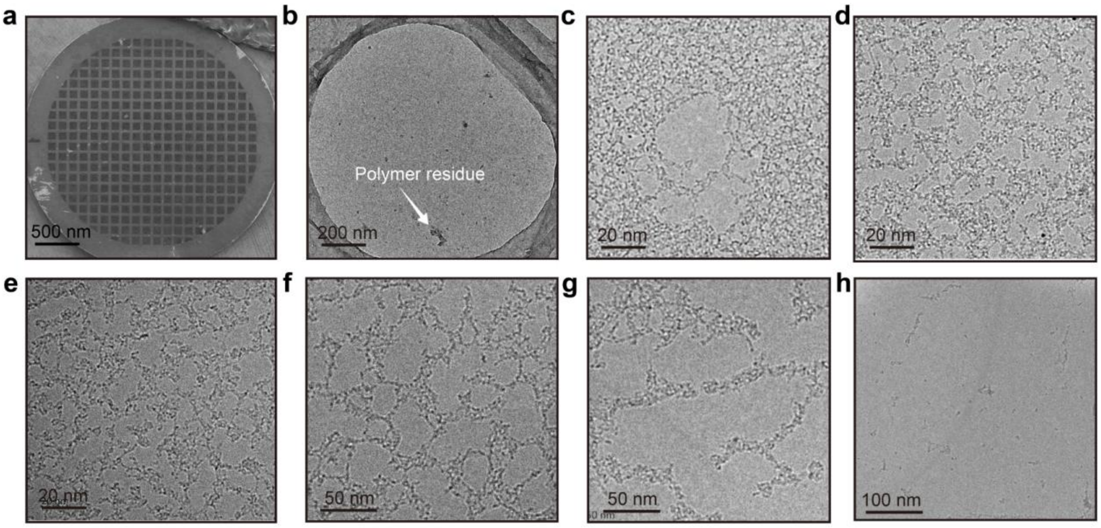

Supplementary Figure 10. The TEM characterization of graphene with different cleanness. a, SEM image of 3-mm sized TEM grid after the transfer of graphene. **b-h**, Representative TEM image with high resolution of graphene membrane with different cleanness, transferred with (**b)**, or without (**c-h**) the assistance of polymer. Note that, if graphene is transferred with the assistance of polymer (panel **b**), no appreciably clean regions (clean region is less than a few nanometres on average) are observed at magnifications similar to that used in panels (**c-h**), since in this case, the graphene surface is significantly contaminated by both transfer-related polymer and amorphous carbon. On the other hand, if graphene is transferred without the use of any polymer, its cleanness is only determined by the presence of intrinsic amorphous carbon. Consequently, we observed that different graphene samples featured different cleanness due to the different amount of amorphous carbon, even though no polymer media were used during transfer.

Supplementary Figure 11: The quantitative measurement of graphene cleanness

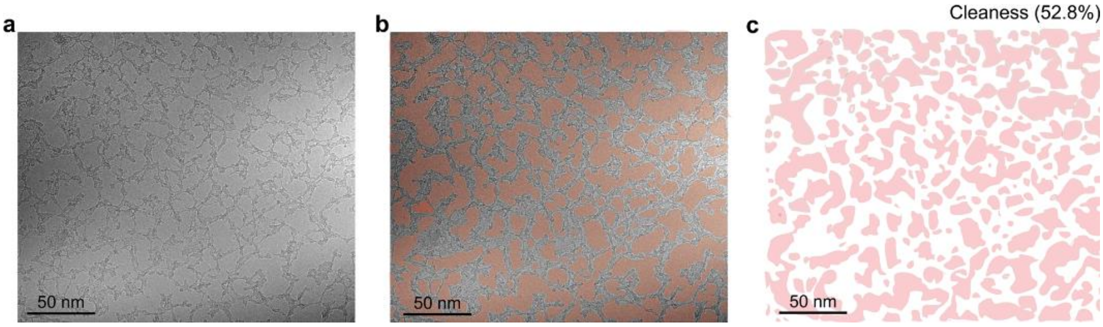

Supplementary Figure 11. Quantitative measurement of graphene cleanness. a, Representative TEM image of graphene surface. **b**, Corresponding false-colored TEM image with clean graphene region marked. **c**, Corresponding image denote the distribution of clean graphene region.

Supplementary Figure 12: Scalable growth of super-clean graphene.

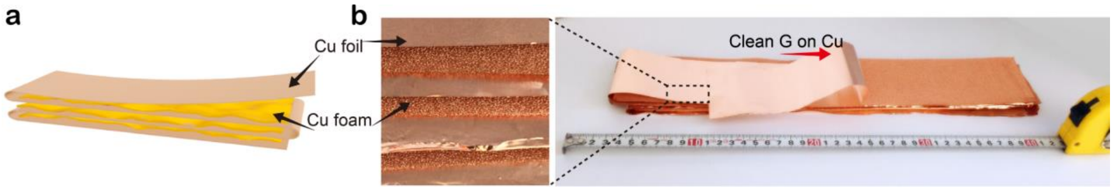

Supplementary Figure 12. Scalable growth of super-clean graphene. Schematic (**a**) and photograph (**b**) of layer-by-layer, foil-foam-stacked structure for growing meter-sized super-clean graphene.

Supplementary Figure 13: Controlling the domain size of super-clean graphene.

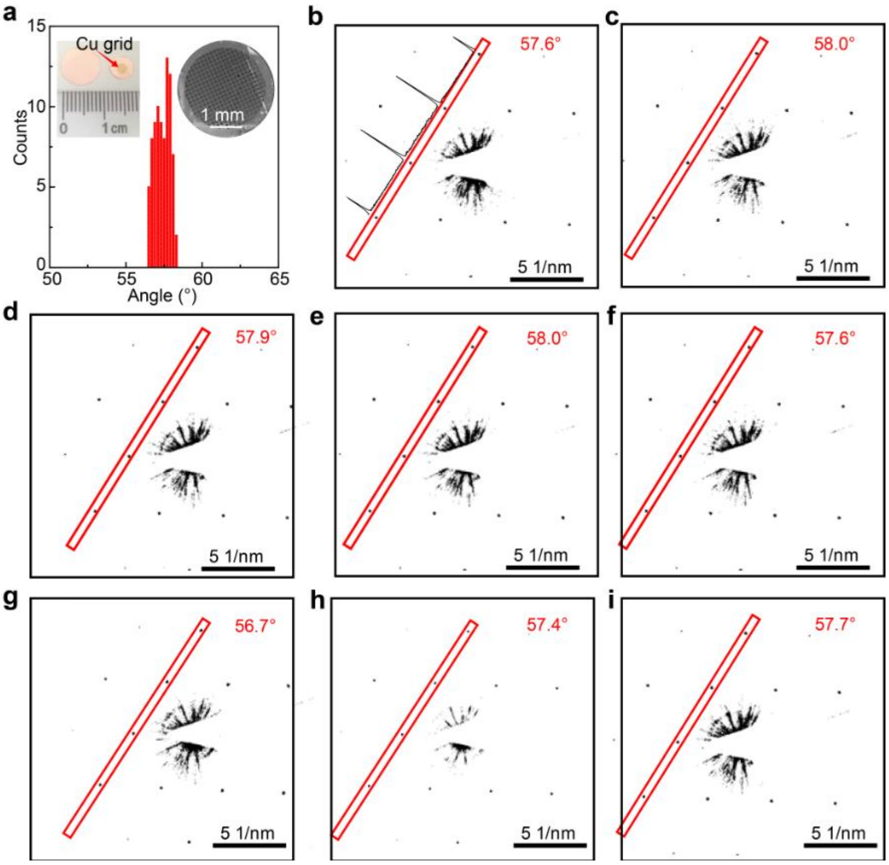

Supplementary Figure 13. Controlling the domain size of super-clean graphene. a, Histogram depicting the angle distribution within the graphene membrane, on a TEM grid, obtained from multiple SAED patterns. The left inset shows punched graphene samples for TiO_2 visualization and the transfer onto the TEM grid. The right inset shows the SEM image of an as-transferred continuous graphene membrane on the TEM grid. **b–i**, Representative SAED patterns collected across the entire graphene membrane. Inset is intensity profiles of the diffraction patterns along the red dashed lines that confirm the monolayer nature of graphene. Note that SAED patterns are obtained from the entire region of graphene on TEM grid (3 mm). The distance between regions where SAED patterns are collected is two square mesh (around 200 μm).

Supplementary Figure 14: The formation and suppression of amorphous carbon.

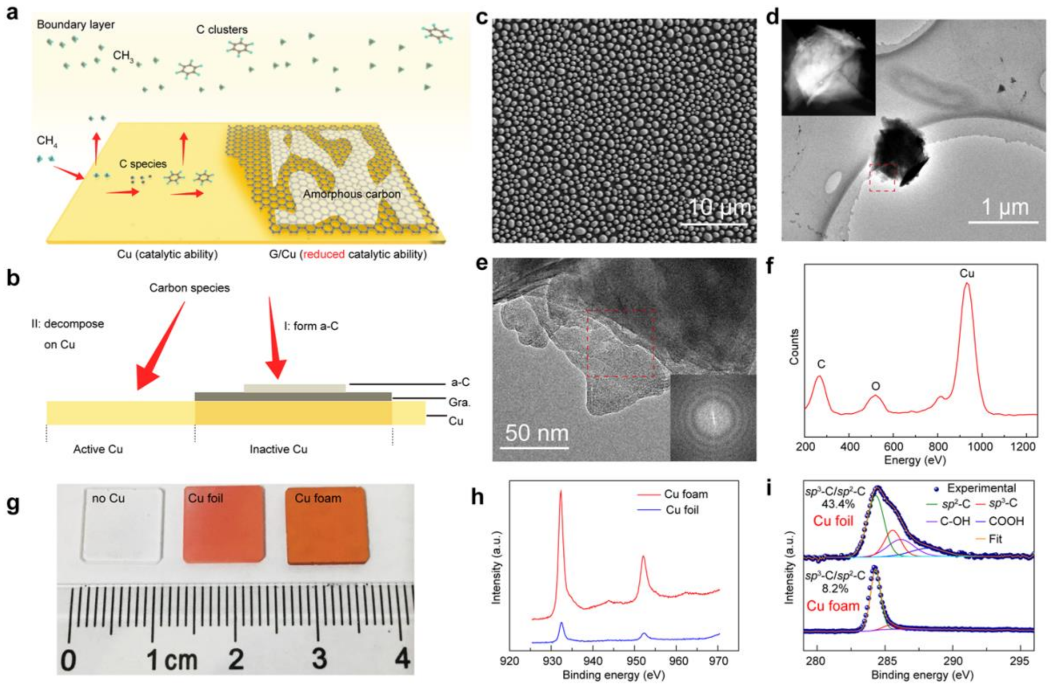

Supplementary Figure 14: The role of Cu clusters in the formation of amorphous carbon. **a**, **b**, Schematics of a possible mechanism for the formation of amorphous carbon (a-C). Note that the inhibited catalytic ability of Cu after being covered by graphene contributes to the formation of graphene on graphene surface. **c**, SEM images of collected Cu clusters on quartz substrate. Note that, CVD-grown graphene on Cu is also used as the collecting substrate, because it can function as the support layer for Cu particles when they are transferred onto the TEM grid for further characterization. **d**, Typical TEM image of a collected Cu nanoparticle. The inset depicts the corresponding HAADF image of the Cu particles. **e**, HRTEM image of the region marked in (**d**). This region is not included on the grid to exclude inference. The inset shows the corresponding Fourier transform of the image marked in (**e**) and indicates the presence of amorphous carbon. **f**, Corresponding EDX spectrum of the region marked in (**e**, **g**). Photograph of quartz substrates after the collection of Cu nanoparticles during growth, when no Cu, Cu foil, or Cu foam is used. **h**, **i**, Cu 2*p* (**h**), and C 1*s* (**i**), core-level XPS spectra of the substrates for the collection of Cu, when Cu foam and Cu foil are used.

Supplementary Figure 15: AFM characterization of the front and back faces of suspended graphene.

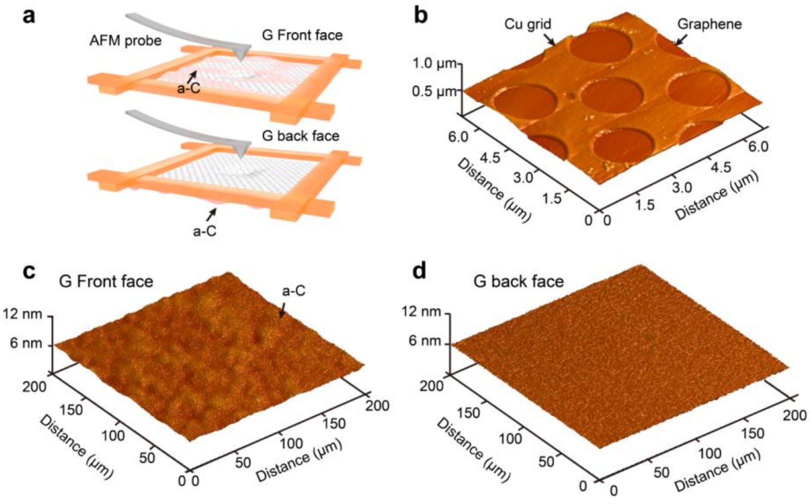

Supplementary Figure 15. AFM characterization of the front and back faces of suspended graphene. a, Schematic of the AFM technique used to probe the presence of amorphous carbon on the graphene surfaces. **b**, Three dimensional (3D)-AFM image of suspended graphene on holey TEM grid. **c**, **d**, 3D-AFM images of the front and back faces of suspended graphene on a holey TEM grid.

Supplementary Figure 16: Comparison of the growth rates of graphene on Cu foil and Cu foam.

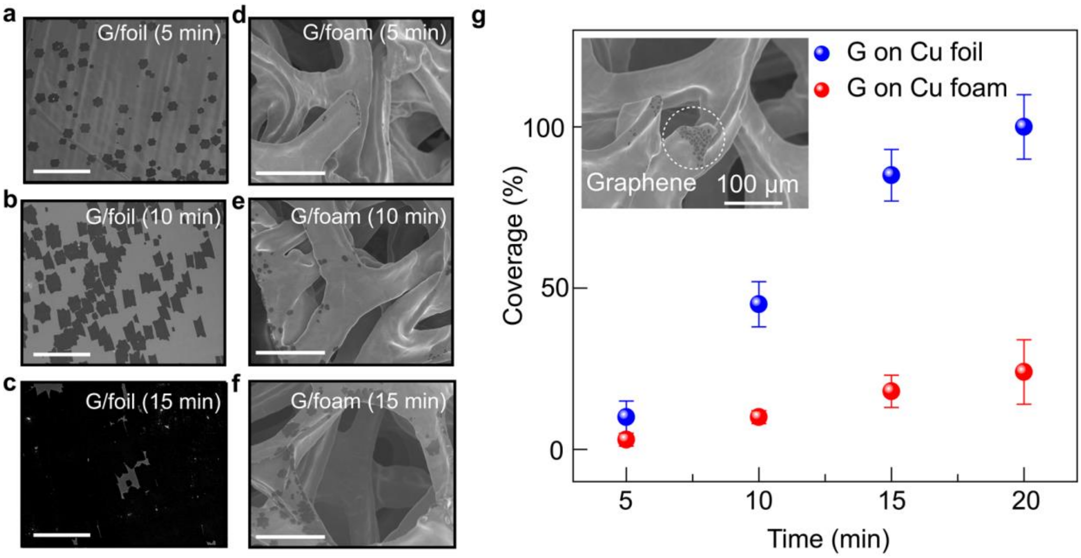

Supplementary Figure 16. Comparison of the growth rates of graphene on Cu foil and Cu foam. (**a**–**f**) SEM images of graphene synthesized within 5 min (**a**), 10 min (**b**), and 15 min (**c**) on Cu foil; and 5 min (**d**), 10 min (**e**), and 15 min (**f**) on Cu foam. **g**, Plot of the graphene coverage on Cu foil (blue) and Cu foam (red), as functions of growth time. Scale bar: 100 μm. The error bar represents the relative deviation.

Supplementary Figure 17: Reduction of transfer-related impurities on the surface of super-clean graphene.

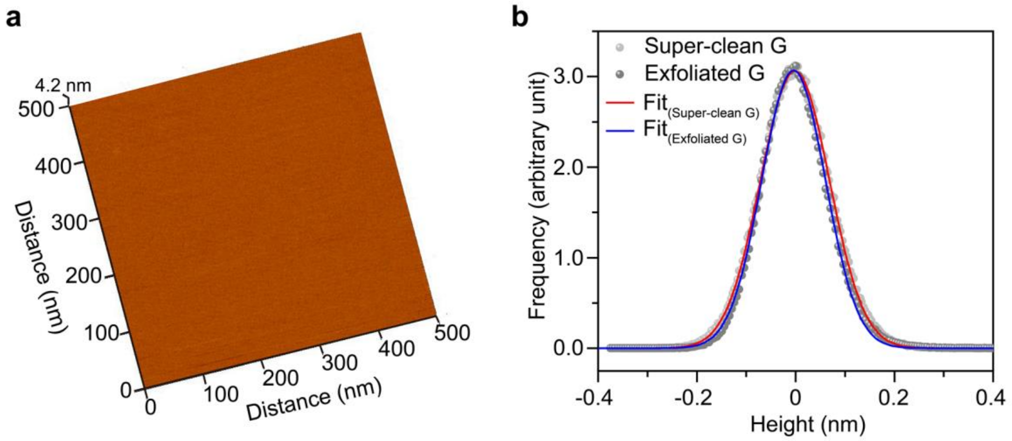

Supplementary Figure 17. Reduction of transfer-related impurities on the surface of super-clean graphene. a, AFM images of transferred super-clean graphene on mica. **b**, Height histograms of super-clean graphene (grey point) and exfoliated graphene (black point) on mica substrate. The data of super-clean graphene is obtained from (**a**).

Supplementary Figure 18: Light transmittance of super-clean graphene.

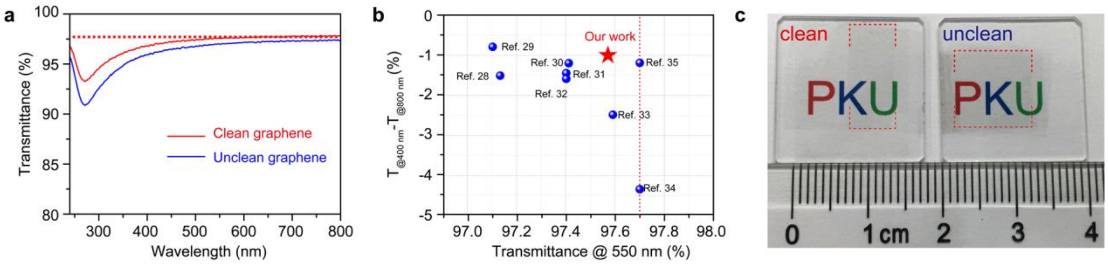

Supplementary Figure 18. Light transmittance of super-clean graphene. a, Ultraviolet-visible (UV-vis) spectra of monolayer super-clean graphene (red) and unclean graphene (blue). **b**, Comparison of graphene transparency values at 550 nm, and reductions of light transmittance from 800 nm to 400 nm in this work and reference. **c**, Photograph of the tri-layer super-clean graphene and unclean graphene samples on quartz substrates, fabricated by layer-by-layer transfer techniques.

Supplementary Figure 19: The selective adsorption of contact metals on amorphous carbon.

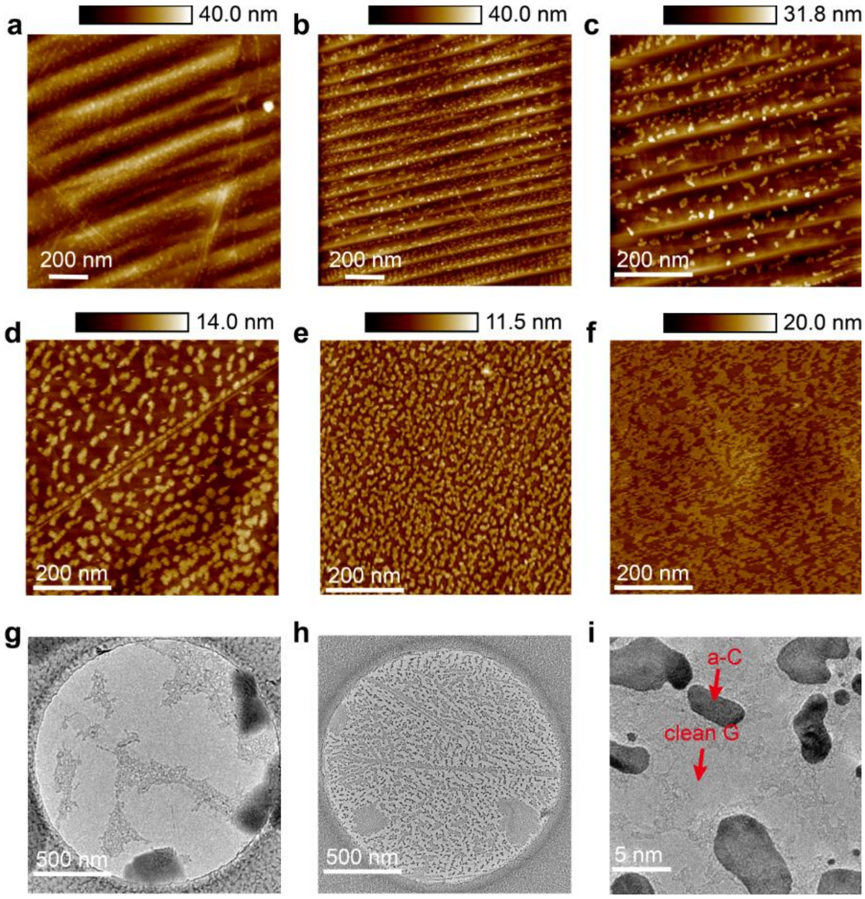

Supplementary Figure 19. The selective adsorption of contact metals on amorphous carbon. a-f, AFM images of graphene on Cu foil after thermal evaporation of Au with a thickness of 0.2 nm. **g**, **h**, TEM images of the Au-decorated graphene membrane with different cleanness after transfer to the TEM grid. **i**, TEM image with high resolution of the region in (**h**). Note that the dark particles correspond to the as-deposited Au.

Supplementary Figure 20: Contact resistance of super-clean graphene using the transfer length method (TLM).

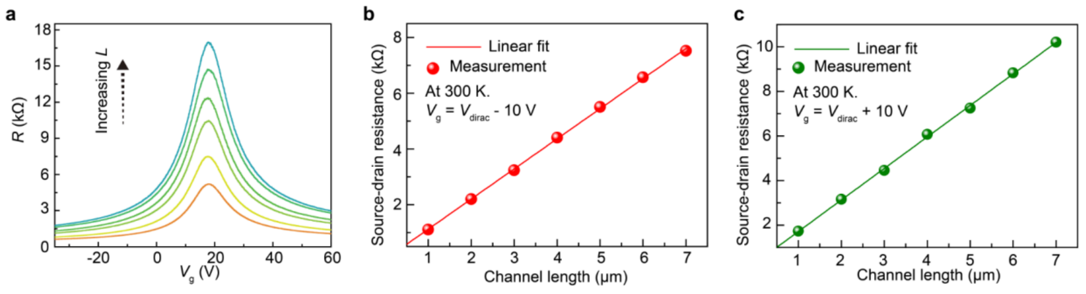

Supplementary Figure 20. Contact resistance of super-clean graphene using TLM. a, Total resistance between the source and drain with increasing channel length as a function of back-gate voltage (V_g). Driving contact resistance using TLM at gate biases of V_{Dirac} - 10 V (**b**), and V_{Dirac} + 10 V (**c**).

Supplementary Figure 21: Reduced contact resistance in super-clean graphene

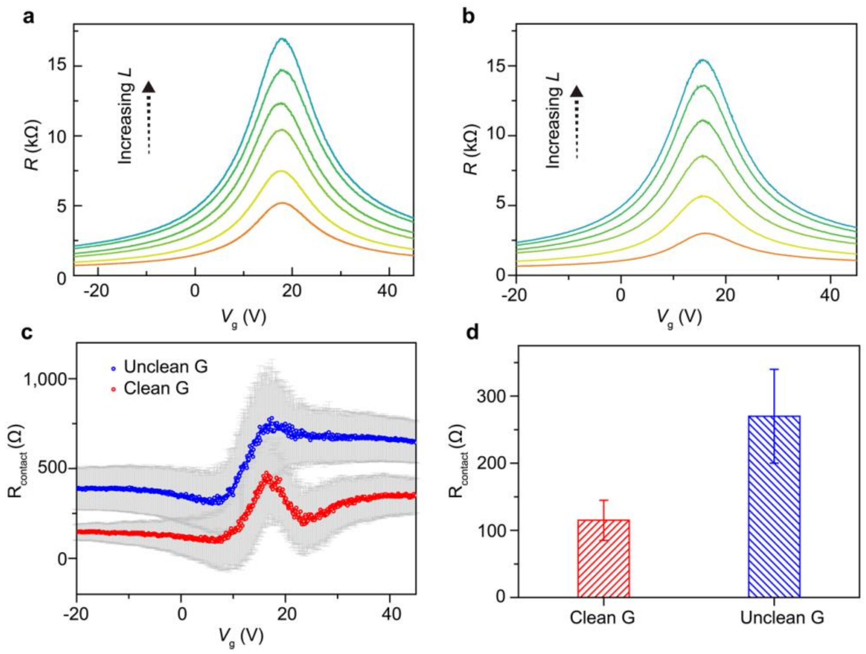

Supplementary Figure 21. Contact resistance comparison between super-clean graphene and unclean graphene surface using TLM. a-b, Total resistance of super-clean graphene (**a**) and unclean graphene (**b**) between the source and drain with increasing channel length as a function of back-gate voltage (V_g). **c**, Measured contact resistance of super-clean graphene (red) and unclean graphene (blue) as a function of gate voltage. The error bar represents the relative deviation. **d**, Statistics of measured contact resistance of super-clean graphene (red) and unclean graphene (blue). Six unclean and six unclean graphene devices are measured for comparison. The error bar represents the relative deviation.

Supplementary Figure 22: Improved carrier mobility of super-clean graphene

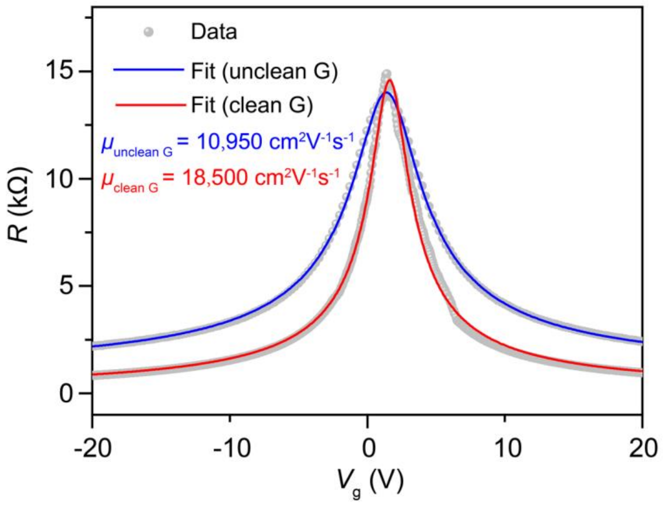

Supplementary Figure 22. Representative transfer curves of clean (red line) and unclean graphene (blue line) on SiO_2/Si substrate.

Supplementary Figure 23: Transport measurement of encapsulated graphene by *h*-BN.

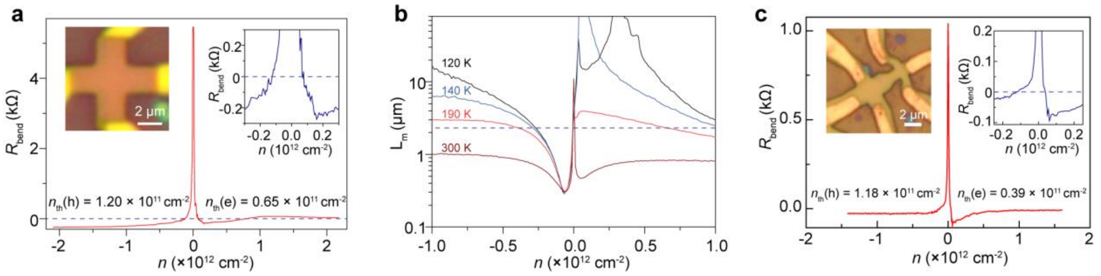

Supplementary Figure 23. Transport measurement of encapsulated graphene by *h*-BN. a, Bend resistance of encapsulated clean graphene measured at 1.7 K. The dash line denotes the zero R_B. Inset (left): Optical microscope image of Hall cross device. Inset (right): Zoom of the plot near the zero R_B. **b**, Mean free path calculated from the diffusive regime as a function of the charge carrier concentration for 120 K, 140 K, 190 K and 300 K. The dash line denotes 2.2 μm. **c**, Bend resistance of encapsulated clean graphene with bar width of 2.5 μm measured at 1.7 K. Inset (left): Optical microscope image of Hall cross device. Inset (right): Zoom of the plot near the zero R_B.

Supplementary Figure 24: Raman characterization of encapsulated graphene by *h*-BN.

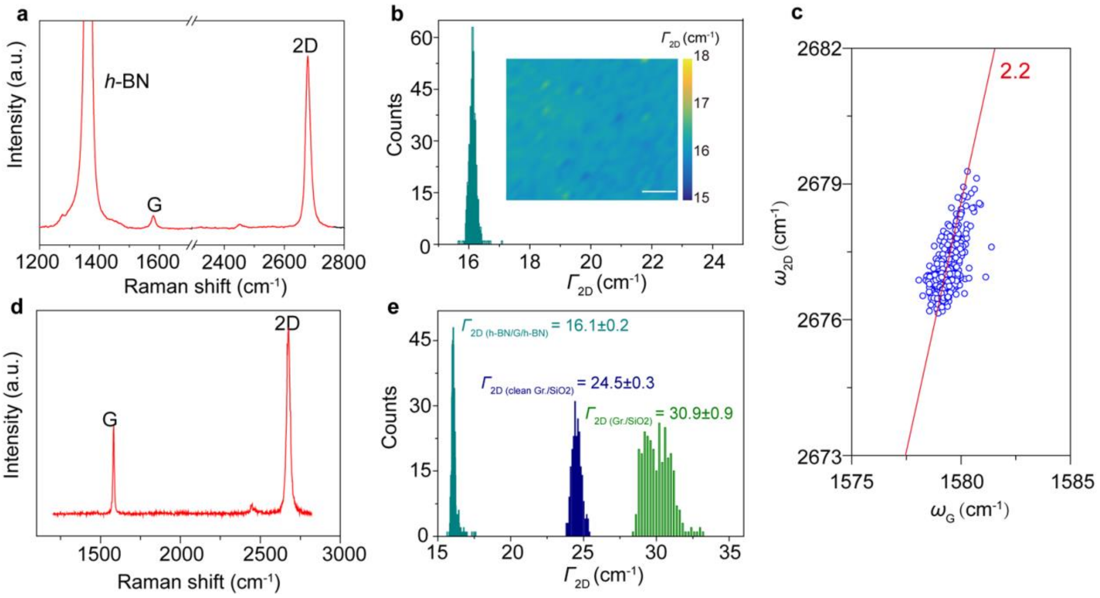

Supplementary Figure 24. Raman characterization of encapsulated graphene by *h*-BN. a, Representative Raman spectrum of graphene encapsulated by h-BN. **b**, Statistics of Γ_{2D}. Inset: Spatial mapping of Γ_{2D} of graphene after encapsulation. Scale bar, 1 μm. **c**, Analyses of Raman spectra (ω_{2D} vs. ω_G) recorded at the same spots in the corresponding mapping. The red line indicates the line with the slope of 2.2. **d**, Representative Raman spectrum of graphene on SiO_2 substrate. **e**, Statistics of Γ_{2D} of encapsulated clean graphene (cyan), clean (navy blue) and unclean graphene (light green) on SiO_2 substrate.

Supplementary Figure 25: Wettability of super-clean graphene.

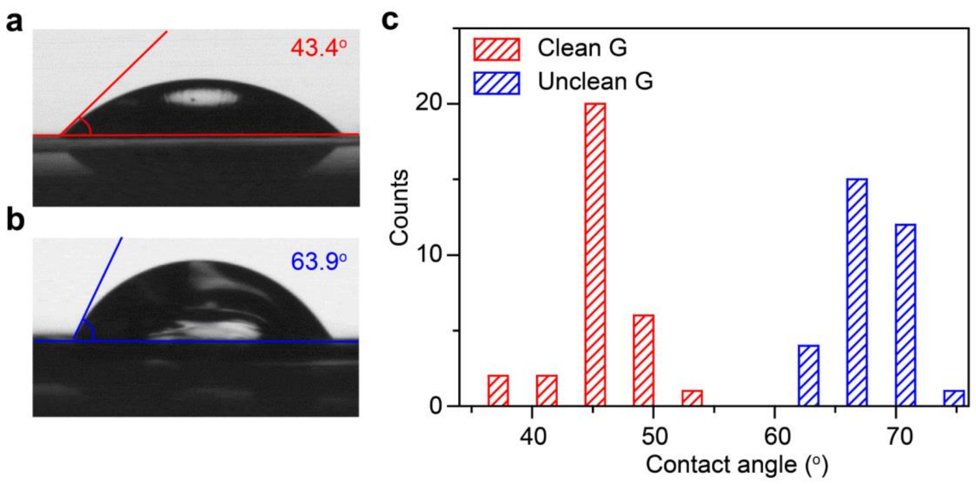

Supplementary Figure 25. Wettability of super-clean graphene. a, b, Representative photographs of the water drops on the surface of clean graphene (**a**) and unclean graphene (**b**). **c,** statistic of the measured WCA for clean graphene (red) and unclean graphene (blue).

Supplementary Figure 26: Thermal conductivity of super-clean graphene.

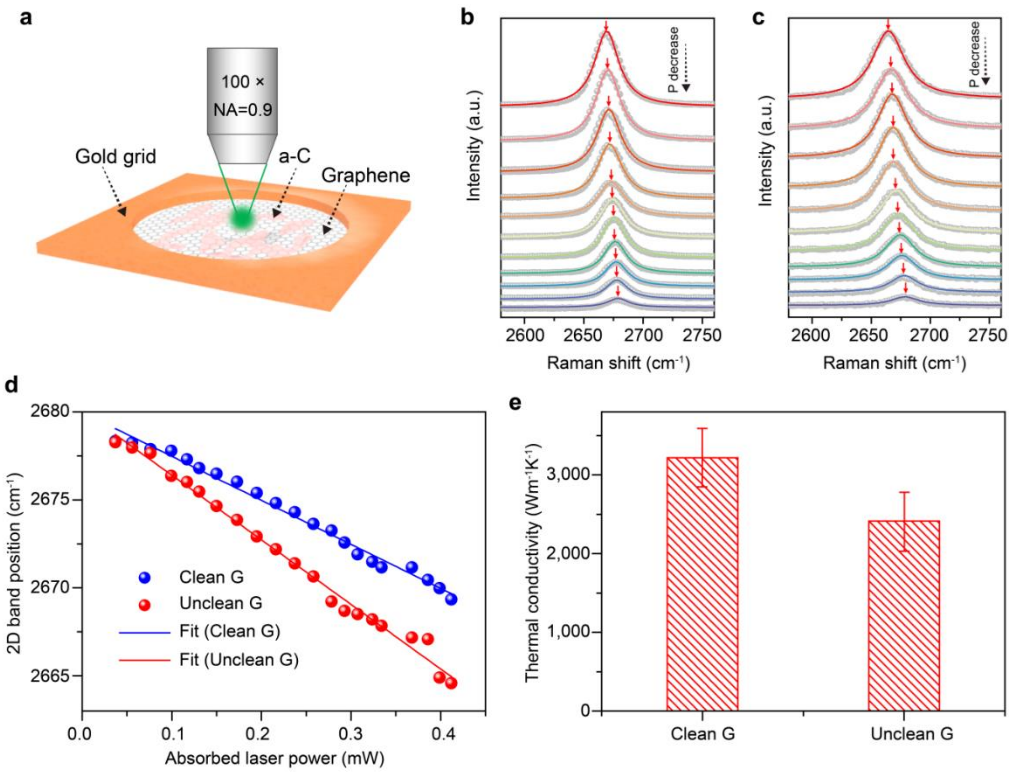

Supplementary Figure 26. Thermal conductivity of super-clean graphene. a, Schematic of the experimental setup for measuring the thermal conductivity of graphene. **b, c,** Raman spectra of suspended super-clean graphene (**b**) and unclean graphene (**c**) excited by 532 nm laser with different power. The values of laser power are 0.037, 0.076, 0.117, 0.150, 0.194, 0.237, 0.278, 0.307, 0.334, 0.386 and 0.412 mW, respectively from bottom to top. **d,** The 2D band shifts measured on suspended graphene as function of the absorbed laser power at room temperature. **e,** Statistics of the measured thermal conductivity for super-clean graphene and unclean graphene. The error bar represents the relative deviation.

Supplementary Figure 27: Reduced sheet resistance of super-clean graphene

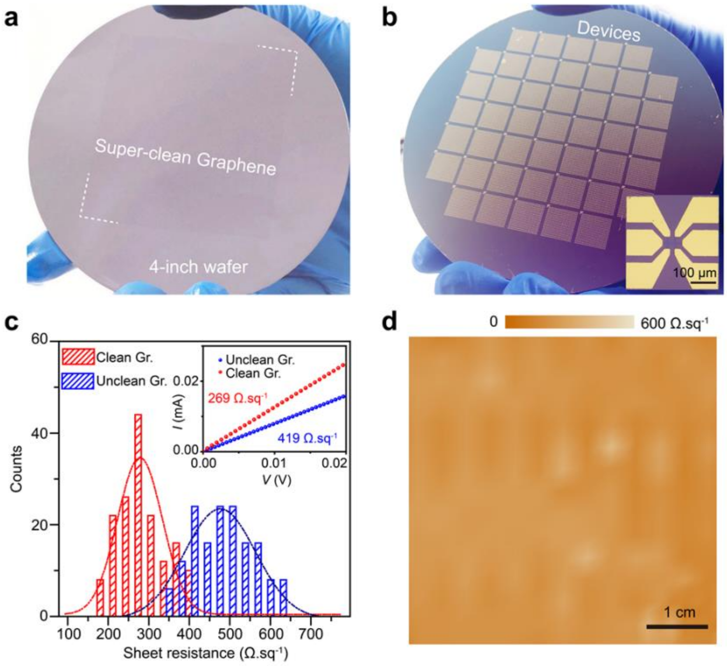

Supplementary Figure 27. Large-scale sheet resistance measurement of clean and unclean graphene. a-b, Contrast-enhanced photograph of the wafer-sized, continuous graphene film on a 4-inch Si/SiO_2 substrate after transfer (**a**) and after the device fabrication. The inset shows OM image of one device structure. Note that there are four types of devices with varying width and length of graphene channel. **c**, statistic of the sheet resistances of clean (red) and unclean (blue) graphene. The inset shows representative *I*–*V* curve of clean (red) and unclean graphene (blue) using a four-probe measurement with channel length and width of 10 and 30 μm, respectively. **d**, Large-scale sheet resistance mapping of super clean with entire size of 10 cm × 10 cm. Note that, there are some regions displaying a relatively high sheet resistance, which might be caused by transfer-induced wrinkle and breakage.

Supplementary Note 1

Discussion for Supplementary Figure 1:

The samples depicted in Supplementary Fig. 1 are from three research groups that also focus on the CVD growth of graphene on Cu foil. The distributions and morphologies of the contaminations on the various graphene surfaces can be visualized by the AFM images (Supplementary Fig. 1), confirming the common presence of amorphous carbon. The height of the amorphous carbon ranges from 0.3 nm to several nanometers. Note that, the area ratio covered by the amorphous carbon on these graphene surfaces is significantly high across all samples. This indicates that surface contamination is a severe problem for CVD-grown graphene. In addition, after growth, graphene on Cu foil usually presents a corrugated morphology that is caused by the difference in thermal expansion coefficients between graphene and Cu[1]. As a consequence, amorphous carbon can only be easily visualized on flat regions where the roughness is less than ~5 nm/μm^2. Otherwise, the roughness of the underlying substrate would interfere with the results.

Supplementary Note 2

Discussion for Supplementary Figure 2:

The presence of surface contamination is clearly visible in the bright field (BF) image and corresponding high angle annular dark field-scanning transmission electron microscopy (HAADF-STEM) image (Supplementary Fig. 2a, b). Furthermore, the carbon and copper exhibited a nearly identical spatial distribution with the contaminations, as evidenced in HAADF-STEM image plus the C and Cu energy dispersive X-ray (EDX) maps (Supplementary Fig. 2a-d and Fig. 2e, f). The EDX spectrum of the contaminated region in the HAADF-STEM image shows C and Cu peaks than those in clean graphene region, confirming the composition of surface contamination (Supplementary Fig. 2g). Further HRTEM image of as-transferred

graphene membrane confirmed the presence of Cu nanoparticles in contaminated region (Supplementary Fig. 2h).

Supplementary Note 3

Discussion for Supplementary Figure 3:

XPS characterization was performed on graphene immediately after growth for elemental analysis to obtain chemical bonding information of specific element in amorphous carbon. The C 1s core-level photoelectron spectrum of graphene on Cu foil displays several peaks, corresponding to sp^2-carbon, sp^3-defecive carbon, C-OH, and COOH bonding (Supplementary Fig. 3a). In detail, the sp^2 peak is related to graphene, while the sp^3 peak is mainly due to the presence of amorphous carbon. Considering the Raman results, amorphous carbon consists of both sp^2- and sp^3-bonded carbon, and would produce a strong D peak in the Raman spectrum (Fig. 1d). In addition, the statistical ratios of sp^2-bonded carbon to sp^3-bonded carbon, from different samples, are displayed in Supplementary Fig. 3b, and clearly show that amorphous carbon commonly exists on graphene surfaces.

Supplementary Note 4

Discussion for Supplementary Figure 4:

Supplementary Fig. 4a shows the TERS and far-field Raman spectra obtained for graphene. The contrast factor (C) is determined according to Supplementary Equation 1:

$$\mathrm{C} = \frac{\mathrm{S_{TERS}}}{\mathrm{S_0}} \qquad (1)$$

Where S_{TERS} is the TERS signal when the tip approached the sample surface, and S_0 is the far-field Raman signal when the tip is retracted.

Consequently, the TERS enhancement factor (EF) can be estimated using the Supplementary Equation 2:

$$\mathrm{EF} = \frac{S_{NF}-S_{FF}}{S_{FF}} \times \frac{A_{FF}}{A_{NF}} = (\frac{S_{TERS}}{S_0} - 1) \times \frac{R_{laser}^2}{R_{tip}^2} \qquad (2)$$

Where A_{FF} and A_{NF} are the areas of Raman signal collected in conventional far-field and TERS experiment and R_{laser} (0.55 μm) and R_{tip} (15 nm) are the radii of the laser focus and the tip, respectively.

Accordingly, we obtain C of 2.5 and EF of 2.02×10^3 for the G peak, and C of 1.7 and EF of 0.94×10^3 for the 2D peak. G peak is more enhanced than 2D peak in TERS because the G peak is located closer to the plasmon resonance peak of the Au tip-Au substrate coupling system. Although we could not obtain the D peak in far-field Raman spectra, we would expect it gives the highest enhancement in all the three vibrational modes as we can observe a considerable TERS signal of D peak.

In addition, the C-H bending peak was also present in the Raman spectrum of contaminated regions, indicating that amorphous carbon might be H-terminated occasionally (Supplementary Fig. 4b). Furthermore, a uniform and high 2D band intensity was observed, indicating a high quality of graphene without intrinsic defects (Supplementary Fig. 4c). TERS intensity profile of D band along one line in the Fig. 1d is presented in Supplementary Fig. 4d, showing a spatial resolution better than 20 nm, which is far beyond the optical diffraction limit. However, we can observe the fine feature smaller than 10 nm, as we can see in the Fig. 1d. Note that, gold tips for scanning tunneling microscopy (STM)-TERS experiments (Premion, 99.998%, 0.25 mm, Alfa)) were prepared with reported method[2].

Supplementary Note 5

Discussion for Supplementary Figure 5:

To further confirm the ubiquity of amorphous carbon on commonly grown CVD samples, we check the graphene samples from three representative graphene research groups, as mentioned in supplementary Fig. 1. The TERS mapping results all exhibited a distribution of a strong D band, confirming the presence of amorphous carbon ((Supplementary Fig. 5).

Supplementary Note 6

Discussion for Supplementary Figure 6:

Both the unclean and super-clean graphene samples were investigated by conventional Raman spectroscopy. In contrast to the TERS results (Supplementary Figure 4 and Supplementary Table 2), the D band intensities in the Raman spectra are near the noise level (Supplementary Fig. 6a-c). Note that these results are in consistent with those in previous works regarding the characterization of graphene samples using conventional Raman spectroscopy (Supplementary Table 1). Consequently, we can conclude that TERS can detect the nanoscale amorphous carbon-related D band, which is usually not detectable in conventional Raman spectroscopy, presumably owing to the considerably higher spatial resolution and selective enhancement of D band intensity than that of conventional Raman techniques3. The three situations can be expected:

(1) In the clean graphene region without the coating of amorphous carbon, no D band would be detectable in TERS (clean region in Fig.1d, red curve) or conventional Raman results4 (dark cyan curve in Fig. 1d).

(2) Due to considerably higher spatial resolution and selective enhancement of D band intensity of TERS[3], we can observe a D band in TERS results (unclean region in Fig. 1d, blue curve; also see the references[5-9]) at the sites of defects or contaminations, while the D band peak could be easily lost in the background in conventional Raman results3 (Supplementary Fig. 6), when the density of defects or amorphous carbon cover a relative small portion of laser focus.

(3) In addition, if the graphene is composed of considerable defects, D band would be observed in both TERS and conventional Raman results, while D band intensity is higher in TERS than that in conventional Raman results[10-13].

Based on above discussion, we can safely rely on TERS to analyse the distribution of amorphous carbon on graphene surface.

Supplementary Note 7

Discussion for Supplementary Figure 7:

^{13}C-graphene was grown using $^{13}CH_4$ (99% purity) as the carbon source, while $^{12}CH_4$ (99% purity) was used for (unlabeled) ^{12}C-graphene growth. After growth, ToF-SIMS measurements were performed on isotopically labeled (^{13}C-graphene) and normal graphene (^{12}C-graphene), to determine the origin of the surface contamination. Negative-ion spectra were collected for each sample. The ToF-SIMS negative-ion spectra of ^{13}C-graphene and ^{12}C-graphene display several peaks: a CH^- ion peak, a CH_2^- ion peak (Supplementary Fig. 7a), and a CH_3^- ion peak (Supplementary Fig. 7b). The CH^- ion peak is produced by graphene, while the CH_2^- and CH_3^- ion peaks originate from the surface contaminants. Clearly, in the ToF-SIMS spectrum of ^{13}C-graphene, the peaks that correspond to amorphous carbon are also found to be isotopically labeled. In addition, each peak is one mass number (*m/z*) heavier that the corresponding ^{12}C-graphene peak. Since the sample can only interact with the ^{13}C isotope ($^{13}CH_4$) during growth, amorphous carbon is simultaneously produced on the graphene surface during graphene growth.

Supplementary Note 8

Discussion for Supplementary Figure 8:

To visualize amorphous carbon on graphene directly in a large scale, the graphene on copper was brought into contact with $TiCl_4$ vapor for the *in-situ* deposition of TiO_2

nanoparticles on the surface, through reaction of $TiCl_4$ and H_2O in humid air. A photograph of the setup for depositing TiO_2 nanoparticles on graphene surfaces is shown in Supplementary Fig. 8a, where misty TiO_2 fog formed in the beaker. The graphene on copper was adhered to the side wall of a Büchner funnel, which was then allowed to contact TiO_2-containing vapor that was passed through the Büchner funnel from the top reaction beaker, to realize a steady stream of TiO_2 fog. This method was also used to visualize ultra-long carbon nanotubes[14]. The unclean graphene surface would adsorb more TiO_2 particles than that of clean one (Supplementary Fig. 8b, c), which is presumably owing to the selective adsorption of TiO_2 particles on amorphous carbon. To prove this, after visualization, the graphene was transferred onto a TEM grid to determine the spatial distribution of the as-deposited TiO_2 on the graphene surface. A typical TEM image of the graphene surface after TiO_2 deposition (Supplementary Fig. 8d) shows darker contrast in the contaminated regions, while clean graphene regions exhibit lighter contrast. In contrast, in HAADF-STEM image, the TiO_2 decorated region displays a bright contrast (Supplementary Fig. 8e). The EDX spectrum of the region with bright contrast shows prominent Ti-related peaks along with a prominent C-related peak in comparison with the clean graphene region, confirming the selective deposition of TiO_2 particles on contaminated region (Supplementary Fig. 8f). Such results were further confirmed by the identical spatial distribution of C, Ti and O EDX elementary maps (Supplementary Fig. 8g-i). Consequently, considering the multi-color nature of TiO_2 particles, the deposition of TiO_2 particles is effective for the direct visualization of amorphous carbon, making them visible to human eyes.

Supplementary Note 9

Discussion for Supplementary Figure 9:

The vertical distances between the Cu foil and foam were kept around 15 μm, as shown in the SEM images (Supplementary Fig. 8a, b). Such narrow gaps, more than

10^5 times smaller than the diameter of the furnace (2.5 cm), ensure a sufficient Cu supply in the small gap, which is important for super-clean graphene growth[15].

Supplementary Note 10

Discussion for Supplementary Figure 10:

Firstly, the as-grown graphene was transferred onto 3-mm-sized holey TEM grid (Supplementary Fig. 10a) using a polymer assisted method[16]. The representative TEM image of as-transferred graphene displays the presence of polymer residue on graphene surface and almost absence of continuous clean graphene region (Supplementary Fig. 10b). To exclude the interference of polymer residues on the determination of cleanness, the no-polymer-assisted transfer was performed. The representative TEM images of graphene membrane with different cleanness were presented in Supplementary Fig. 10c-h. Note that, the region with bright contrast corresponds to the clean graphene region.

Supplementary Note 11

Discussion for Supplementary Figure 11:

Firstly, in this work, we measured quantitatively the cleanness of graphene surface using TEM image (Supplementary Fig. 11a). The definition of the cleanness is the ratio of the area of clean graphene region to entire imaging region. In TEM image, the clean graphene region displays a uniform brighter contrast while the contaminated graphene region indicated a darker contrast, as confirmed by the high-resolution TEM images. We measured the area of the clean graphene region, and then calculated the ratio of the clean area to entire area (Supplementary Fig. 11b, c). In this case, the cleanness of Supplementary Fig. 11a is around 52.8%, accordingly.

Supplementary Note 12

Discussion for Supplementary Figure 12:

Further folding of the copper foil, to form an alternating, layer-by-layer, foil-foam-stacked structure, promises the scalable growth of super-clean graphene on Cu foil, as schematically illustrated in Supplementary Fig. 12a. In addition, the photograph of layer-by-layer structure before the growth is presented in Supplementary Fig. 12b.

Supplementary Note 13

Discussion for Supplementary Figure 13:

The grain size of single-crystal graphene can be enhanced by using smaller molar ratios of methane to hydrogen (carbon source supply)[17]. In this manner, a continuous graphene film, consisting of millimeter-sized graphene single crystals, can be successfully synthesized. To determine the domain size of the graphene membrane, the graphene on copper foil was sampled at different locations by punching, and the punched samples were subsequently transferred onto the TEM grids (Supplementary Fig. 13a, inset). Statistical data on the relative angles of the graphene-lattice directions (Supplementary Fig. 13a), extracted from the selected area electron diffraction (SAED) patterns (Supplementary Fig. 13b-i), reveal relative rotation angles of less than ±2.0°, confirming the single-crystal nature of graphene over a 3-mm-sized region (size of TEM grid)[18]. We can conclude that the domain size of the graphene produced is at least 3 mm.

Supplementary Note 14

Discussion for Supplementary Figure 14:

A possible mechanism for the formation of amorphous carbon is illustrated schematically in Supplementary Fig. 14a. Firstly, the decomposition of methane results in the dense production of CH_3 species on the Cu surface[19]. Subsequently, some of these radicals are desorbed from the Cu surface, while others undergo further dehydrogenation to form C clusters, as graphene precursors[20]. The presence of C clusters in the initial stage of graphene growth was also experimentally confirmed in previous work[21]. The C clusters with the higher binding energies between the C edges and the Cu substrate are more stable and fuel the subsequent growth of graphene, while unstable clusters, with lower binding energies, detach from the surface into the boundary layer. These C-H species would undergo the decomposition, which is catalyzed by Cu, when deposited on Cu surface[19]. In contrast, C-H species would fuel the formation of amorphous carbon on graphene surface, because catalytic ability of the underlying Cu is inhibited by the coverage of graphene, which was widely reported (Supplementary Fig. 14 a, b)[22].

As the temperature is close to the melting point of Cu, the intense evaporation of Cu also results in a high density of Cu clusters in the boundary layer[23]. Interestingly, we observed amorphous carbon would cover the Cu nanoparticles collected. The SEM image shows the morphologies of the collected Cu nanoparticles (Supplementary Fig. 14c). After transferring the collected Cu nanoparticles onto a TEM grid (Supplementary Fig. 14d), HRTEM (Supplementary Fig. 14e) reveals that amorphous carbon with characteristic Fourier transform results cover the Cu nanoparticle. The presence of amorphous carbon is also evident in the EDX spectrum (Supplementary Fig. 14f).

The formation of amorphous carbon on the graphene surface is caused by the highly inhibited catalytic ability of the underlying Cu. Note that similar structure in enclosed systems (Cu envelop or stacking Cu foil), the Cu vapor supply can be improved in comparison with that of conventional Cu foil, which could also help in the enhancement of cleanness. However, in such structure, where Cu foil itself functions

as the growth substrate, graphene would grow on two sides of the Cu foil quickly. Thus, the Cu vapor supply would still significantly reduce, especially when graphene almost fully covers the Cu foil. In contrast, the Cu foam, due to its unique porous three-dimensional structure and higher specific surface area, would provide sufficient Cu vapor during the growth, thus enabling the decomposition of carbon species and amorphous carbon. Furthermore, the growth rate of graphene on Cu foam is significantly lower than that on conventional Cu foil (Supplementary Fig. 16a-g), thus Cu foam would continuously supply Cu vapor without being influenced by the coverage of graphene on Cu foil. Thus due to high specific surface area and a lower growth rate of Cu foam, the Cu foam in the vertical stacking structure promises a continuous supply of Cu vapor during the entire growth of graphene on Cu foil to enhance the final cleanness.

To confirm the sufficient Cu vapor supply using Cu foam, quartz substrates were used to collect the Cu during graphene growth, when either Cu foil or Cu foam was used. After the deposition of Cu nanoparticles, the quartz substrate displays a deeper orange color when Cu foam is used, than when Cu foil is used (Supplementary Fig. 14g). These results show that more nanoparticles are deposited onto the substrate when Cu foam is used, which is also evident in higher Cu signals in the XPS spectrum (Supplementary Fig. 14h). The highly inhibited formation of amorphous carbon is also evident in the XPS analysis of the carbon on the collected Cu nanoparticles in boundary layer. In detail, the intensity ratio of the defective sp^3 peak to sp^2 carbon peak is reduced when Cu foam is used, indicating that less defective amorphous carbon is produced (Supplementary Fig. 14i). Furthermore, as evident in the Raman results, the formation of amorphous carbon on Cu nanoparticles is highly reduced when Cu foam is used, enabling the formation of graphene on Cu nanoparticles (Fig. 2f). In addition, Cu foam, with stronger adsorption capacity, would be expected to absorb more carbon species than Cu foil, resulting in the reduced formation of amorphous carbon on graphene/Cu foil.

Supplementary Note 15

Discussion for Supplementary Figure 15:

Further evidence in support of the transport of amorphous carbon from the boundary layer to the graphene surface is provided by the presence of amorphous carbon on the front face of graphene, the surface that is not in contact with the Cu substrate (Supplementary Fig. 15a). To confirm this, atomic force microscopy (AFM) analyses were conducted on the front and back faces of the suspended graphene, which was fabricated by the non-polymer assisted transfer of graphene onto a holey TEM grid (Supplementary Fig. 15b). Because of the extremely high flexibility of suspended graphene, amorphous carbon can only be detected when the AFM probe directly contacts it; otherwise, the interaction force exerted by the AFM probe on the graphene during scanning causes graphene fluctuations, in turn masking the signals produced by the amorphous carbon. The AFM image of the front face of graphene, which does not contact the Cu substrate, shows the distinct presence of amorphous carbon (Supplementary Fig. 15c). In contrast, amorphous carbon is invisible in the AFM image of the back face of graphene (Supplementary Fig. 15d), confirming that no amorphous carbon exists on this back face. Consequently, amorphous carbon, which is produced from the carbon species in the boundary layer, is directly deposited on the front face of graphene, thereby contaminating the surface.

Supplementary Note 16

Discussion for Supplementary Figure 16:

To quantitatively obtain the growth rates of graphene on Cu foam and Cu foil, we took ex-situ SEM images of graphene on Cu foam and Cu foil at specific growth times (Supplementary Fig. 16a-f). In detail, at time *t*, the carbon supply source (0.5 sccm CH_4) was interrupted and Ar was immediately allowed to flush into the chamber

at a rate of 200 sccm. We found that graphene fully covers the Cu foil within 20 min, while only some isolated graphene grains are formed at the edges and steps of the Cu foam, with less than 25% coverage (Supplementary Fig. 16g). Note that, the definition of the growth rate is the time-dependence of the coverage of graphene on Cu foam and foil. Due to higher specific surface area of Cu foam than that of Cu foil, graphene needs a very long time to fully cover Cu foam (exceeding 3 h) than that on Cu foil (20 min) at the same growth parameter. It should be mentioned that the distribution of the graphene grains especially at low-pressure CVD system (LPCVD) is non-uniform. The graphene grains prefer to be formed at the bulge regions (uneven regions), thus leaving other places uncovered to continuously supply Cu vapor (Supplementary Fig. 16g, inset). Consequently, during the entire growth of graphene on Cu foil, the Cu foam remains mainly uncovered, providing a continuous supply of sufficient Cu vapor for suppressing contamination.

Supplementary Note 17

Discussion for Supplementary Figure 17:

Note that the very small fluctuation in Fig. 3b is caused by small fluctuation of the substrate, as evidenced from the similar height histogram of super-clean graphene and exfoliated graphene (Fig. 3c). Such a small fluctuation is not observed after the transfer of super-clean graphene on to the atomically flat mica substrate (Supplementary Fig. 17a, b).

Supplementary Note 18

Discussion for Supplementary Figure 18:

To evaluate the light transmittances of super clean and unclean graphene, the graphene samples were transferred onto quartz substrates with the assistance of poly(methyl methacrylate) (PMMA). The layer number of as-grown graphene film

was carefully controlled in CVD growth to exclude its influence on transmittance. Note that, the transfer-related breakage and wrinkles were taken into consideration in the measurement of light transmittance of graphene, to ensure the reliability of our measurements. A clear enhancement of light transmittance was observed in our clean sample (~ 97.6% at 550 nm) than that of unclean counterpart (~ 97.0% at 550 nm) (Supplementary Figure 18a), after taking into consideration of layer number and wrinkles. As reported previously[24], the reduction of polymer residues on graphene surface contributes to the enhancement of light transmittance along with the elimination of amorphous carbon. Indeed, the theoretic light absorption of graphene is $\pi\alpha \approx 0.023$ in visible region. However, even light absorption of mechanically exfoliated graphene is not a constant value[25], where intrinsic light absorption of monolayer graphene is actually higher than theoretical value ($\pi\alpha$) at 550 nm and increases gradually from infrared region to visible and ultraviolet region. This additional absorption is attributed to presence of an excitonic resonance absorption peak at around 270 nm induced by the van Hove singularity at the saddle point of the band structure (M point)[24,26,27]. Previously presented Fano model can be used to quantitatively describe the relevant experimental data of graphene absorption, clearly confirming the absorption value at 550 nm is relatively higher than $\pi\alpha$[24,26,27]. In addition, the presence of polymer residues would broaden this absorption peak, which would induce a further reduction of light transmittance form infrared region to visible region[24,25]. In that case, reduction of light transmittance from 800 nm to 400 nm can be used to reflect the presence of polymer residue[25]. A small reduction of light transmittance from 800 nm to 400 nm in clean graphene in turn indicates a reduction of polymer residue (Supplementary Fig. 18b). A lighter contrast in super-clean graphene due to the enhanced transmittance becomes more prominent in multilayer graphene fabricated by a layer-by-layer transfer technique (Supplementary Fig. 18c).

Supplementary Note 19

Discussion for Supplementary Figure 19:

To probe the deposition behavior of contact metals, Au and Cr were slowly evaporated onto freshly grown unclean graphene on Cu foil, up to a thickness of 0.2 nm, using a thermal evaporator at 0.1 Å/s under a vacuum of 10^{-5} Pa. The AFM images of the graphene surface decorated by Au particles (Supplementary Fig. 19a-f) shows that the deposition of the metal is not uniform over the graphene surface; it is rather similarly distributed to the amorphous carbon presented on the graphene surface. To confirm this relationship, we transferred the metal-decorated graphene onto a TEM grid. The TEM images of the graphene membrane show that the spatial distribution of the Au particles is unchanged, and shows no obvious coalescence. In addition, the graphene with different cleanness also display different spatial distribution of Au particles (Supplementary Fig. 19g, h). The HRTEM image of the graphene membrane clearly confirms the selective deposition of Au particles onto the contaminated region; the Au particles are always absorbed onto the contaminated regions with darker contrast (Supplementary Fig. 19i). This was also observed in previous work[36,37,]. This deposition behavior results in smaller grain sizes of the deposited metal, and less effective contact area between the metal and graphene. As a consequence, the contact resistance of unclean graphene would be larger than that of the super-clean analog.

Supplementary Note 20

Discussion for Supplementary Figure 20:

TLM was used to extract the contact resistance of super-clean graphene. The resistance between the source and drain of the TLM devices (Fig. 3c, inset) with increasing channel length as a function of back-gate voltage (V_g) is presented in Supplementary Fig. 20a. Typical results at $V_g = V_{Dirac} - 10$ V and $V_g = V_{Dirac} + 10$ V, respectively, are presented in Supplementary Fig. 20b-c. The fitted contact resistances are 105 Ω·µm ($V_g = V_{Dirac} - 10$ V), and 230 Ω·µm ($V_g = V_{Dirac} + 10$ V).

Supplementary Note 21

Discussion for Supplementary Figure 21:

To provide a more convincing comparison of contact resistance, we carefully measured the contact resistance of super-clean graphene and unclean graphene with similar work function (similar Dirac point positon in the transfer curves) using the transfer length method (TLM) (Supplementary Fig. 21a, b). The contact resistance of clean graphene (115 ± 23 Ω μm with minimum value of 92 Ω μm) was lower than that of unclean graphene (351 ± 36 Ω μm) (Supplementary Fig. 21c, d). In addition, there are many previous works on the reduction of polymer residue to achieve a better contact between graphene and metal, through plasma treatment[38,39], laser cleaning[40], CO_2 cluster cleaning[41], and ultraviolet-ozone treatment[42]. Therefore, we can conclude that the availability of super-clean graphene surface guarantees a lower contact resistance.

Supplementary Note 22

Discussion for Supplementary Figure 22:

To confirm the enhancement of carrier mobility in super-clean sample, we have carefully measured the carrier mobility of clean and unclean graphene, based on 12 × 2 devices, respectively. The corresponding results are shown in Supplementary Fig. 22 and Supplementary Table 6, where average carrier mobility of clean graphene (17,000 $cm^2 V^{-1} s^{-1}$) is clearly higher than that of unclean counterpart (11,000 $cm^2 V^{-1} s^{-1}$). Note that, the fitting is based on nonlinear fitting method[18]. The enhanced carrier mobility is presumably owing to reduction of polymer residue, which was reported to result in a reduced carrier mobility and a larger fluctuation range of carrier mobility results[43]. Note that, graphene devices would exhibit a further enhanced mobility through encapsulation by *h*-BN, due to the reduced carrier inhomogeneity and reduced intrinsic doping in comparison with SiO_2/Si-supported devices[44,45].

Supplementary Note 23

Discussion for Supplementary Figure 23:

Many factors would influence the carrier mobility of graphene such as surface contamination, substrate roughness and scattering, and transfer-related doping. To further increase the carrier mobility of clean graphene and exclude the influence of substrate scattering and doping, we have fabricated the encapsulated graphene samples, according to reported "pick-up" techniques[45,46].

For understanding the ballistic transport behavior of clean graphene, the encapsulated graphene was patterned by electron beam lithography and reactive ion etching into standard Hall cross structure (Supplementary Figure 23a, inset)[47]. The basic approach to probe the ballistic transport is to measure bend resistance, R_B, and wiring scheme is similar to previous reference[47]. Supplementary Figure 23a shows the R_B of the measured devices at the low temperature (1.7 K). The charge neutrality point can be identified by peak position of R_B, which is around 0.04×10^{11} cm^{-2}, indicating a clean device with reduced doping level. The R_B becomes negative for both the electron and hole doping. Similar results have been observed in different electrodes. Thus, the negative bend resistance confirms the ballistic transport of charge carriers from one contact to its opposite voltage contacts without being scattered. We can safely conclude that carrier in this device exhibits an elastic mean free path (l_m) large than the width of Hall cross structure (2.2 μm). The accurate calculation of l_m can be performed from the van-der-Pauw conductivity through the Supplementary Equation 3:

$$l_m = \sigma h / 2e^2 k_F \qquad (3)$$

where $k_F = (\pi n)^{1/2}$ in the diffusive transport regime and n is carrier density) (Supplementary Figure 23b). Consistent with references[46,48], there is an overall increase of l_m at the regions with a larger carrier density at different temperatures.

As indicated, the l_m would reach 2.2 μm at low temperature when the transport become ballistic, while at room temperature the l_m is around 1 μm, consistent with the results of exfoliated graphene[47,48] (Supplementary Figure 23b). We can use the threshold carrier density (n_{th}) in which the system enters the ballistic transport regime to estimate the carrier mobility, according to the Supplementary Equation 4:

$$\mu = 2e\pi^{1/2} l_m / \left[h (n_{\text{th}})^{1/2}\right]^{46} \qquad (4)$$

The n_{th} for holes is $1.20 \times 10^{11}\ \text{cm}^{-2}$ and n_{th} for electrons is $0.65 \times 10^{11}\ \text{cm}^{-2}$, if the we use the l_m = 2.2 μm, the carrier mobility is calculated to be 510,000 $cm^2\ V^{-1}\ s^{-1}$ for hole side and 750,000 $cm^2\ V^{-1}\ s^{-1}$ for electrons.

In another device with the bar width of 2.5 μm, the n_{th} for holes is 1.18×10^{11} cm^{-2} and n_{th} for electrons is 0.39×10^{11} cm^{-2}. If the we use the l_m = 2.5 μm, the carrier mobility is calculated to be 625,000 $cm^2\ V^{-1}\ s^{-1}$ for hole side and 1,083,000 $cm^2\ V^{-1}\ s^{-1}$ for electrons side (Supplementary Figure 23c).

There are four reasons for the improved carrier mobility in encapsulated graphene: (1) Reduced transfer-related doping: the "pick-up" techniques for encapsulating graphene can avoid the contact of graphene with water, which can dope the graphene and impede its quality; (2) Reduced roughness and substrate scattering on *h*-BN substrate than SiO_2; (3) No polymer residues: the transfer media is *h*-BN itself, rather than the polymer, thus avoiding polymer residue on graphene surface; (4) Enhanced cleanness due to movement of contamination at the interface: according to previous reports[50], contaminants on graphene surface tend to diffuse at the *h*-BN-graphene interface. In this case, the moving contamination commonly forms relatively large (μm-sized) pockets of trapped material, which are clearly seen as "bubbles" in optical microscopy images[50]. In the device fabrication, graphene regions without such bubbles (clean regions), were carefully chosen to ensure a better cleanness. In our case, the improved

carrier mobility of graphene on SiO_2 is caused by the reduced transfer-related and amorphous carbon[43]. In encapsulated graphene, the availability of clean interface in our case can also contribute to improved carrier mobility along with the contribution of squeezing effect of *h*-BN to the improved cleanness. These two kinds of devices are both important for graphene research and applications[51]. Encapsulation routine sometimes requires tedious sample fabrication processes and high-quality *h*-BN substrates with enough size for large-area fabrication and applications of CVD graphene[46]. Consequently, graphene-on-SiO_2 devices are still one of predominantly used for electrical applications to date. Therefore, the efforts devoted to optimizing transfer techniques (for example, obtaining large-area high-quality *h*-BN and suppressing the formation of bubble) and improving cleanness of graphene on different substrates are both important for the future applications of graphene devices.

Supplementary Note 24

Discussion for Supplementary Figure 24:

For the Raman results of graphene, a low value of full width at half maximum of 2D peak (Γ_{2D}) is a hallmark of high carrier mobility[46,52.] After the encapsulation, a sharp and uniform distribution of Γ_{2D} with a lower average value of ~16.1 cm^{-1} was observed for our encapsulated samples (Supplementary Figure 24 a and b), which is comparable with those reported in other high-mobility samples[45,53,54]. As shown in Supplementary Figure 24 a, the wavenumbers of G and 2D peak maxima were 1580 cm^{-1} and 2678 cm^{-1}, respectively, indicative of a reduced doping level.

In addition, a lower value of Γ_G (~12 cm^{-1}) was observed along with reduced Γ_{2D}. The Γ_G and Γ_{2D} are expected to be influenced by both the nanometer-scale strain variation and doping effects. Furthermore, we found that the area of the 2D peak does not depend on Γ_{2D}, which allows one to dismiss the doping effect as the main factor of influence for Γ_{2D}[53,54]. Thus, the boarding of 2D and G peaks can be ascribed to local

structural deformation. A reduced Γ_G and Γ_{2D} in our samples is indicative of reduced local strain on the graphene lattice, which might contribute to higher carrier mobility. In addition, in agreement with previous results[53,54], the positions of G (ω_G) and 2D (ω_{2D}) peaks were scattered around a line with a slope of 2.2, which is coincident with the ratio of strain-induced shifts (Supplementary Figure 24 c). The narrow spread of the data points in our sample translates into a very low maximum micrometre-scale variation of ~0.083%, which is very close to the value of pristine graphene with a reduced doping level reference[54]. In addition, we found that clean graphene on SiO_2 substrates using the "dry transfer" method also exhibited a Γ_{2D} value (~24.5 cm^{-1}) lower than those of common samples (30.9 cm^{-1}) and a reduced doping level ($\omega_G \approx$ 1582 cm^{-1}), which further confirmed the improved carrier mobility of the former (Supplementary Figure 24 d and e). Consequently, we believe that the results of Raman spectroscopy analysis confirm that our clean sample exhibited reduced structural deformation and reduced doping level, providing a proof of high carrier mobility and quality.

Supplementary Note 25

Discussion for Supplementary Figure 25:

Water contact angles (WCAs) of super-clean graphene and commonly grown unclean graphene on Cu foil were measured immediately after growth to characterize the wetting proprieties difference caused by the presence of surface contamination. Note that we surveyed an area of 2 cm × 2 cm for obtaining an average WCA for a certain sample. The representative photographs of water drops captured on clean and uncleans graphene surface are presented in Supplementary Fig. 25a, b, indicating a clear difference in wetting properties. Such difference was further confirmed by the statistics of measured WCA, where average WCA for clean graphene is around 45° and for unclean samples the corresponding value is around 67° (Supplementary Fig. 25c). It has been widely accepted that surface contamination would strongly influence

the surface properties, especially the wettability of graphene[55]. Consequently, the super-clean graphene sample, almost devoid of surface contamination, would become more intrinsically hydrophilic than unclean counterpart.

Supplementary Note 26

Discussion for Supplementary Figure 26:

The thermal conductivity of graphene was measured according to previous reported non-contact Raman optothermal method[56] based on measuring the Raman spectra of suspended graphene, which is fabricated by the transfer of graphene onto an Au grid. In detail, during the measurement, a 532 nm laser beam is focused on the center of the suspended graphene (4.0 μm diameter) through 100 × objective lens with a numerical aperture (NA) of 0.9 (Supplementary Fig. 26a). The optical absorption of graphene is measured to be around 4.2 ± 0.5% using a homemade microabsorption spectrometer that has a spatial resolution of <3 μm. Both the thermal conductivity of super-clean graphene and commonly grown unclean sample were probed. The temperature rise in the optically heated graphene membrane by laser beam causes a clear red-shift of the 2D band in obtained Raman spectra. In turn, the 2D peak position can be used to reflect the temperature of graphene heated by the laser at different powers. Supplementary Fig.26b, c display the Raman spectra of super-clean graphene and unclean graphene, respectively, excited by laser at different power, showing a clear red shift of 2D band with increasing Raman laser power. The relationship of the obtained 2D band position (ω) and absorbed power (P) on sample was shown in Supplementary Fig. 26d. Consequently, the thermal conductivity of suspended graphene can be estimated according to the Supplementary Equation 5:

$$\kappa = \frac{\chi_{2D} \ln\frac{R}{r_0}\alpha}{2\pi t\ \delta\omega/\delta P} \qquad (5)$$

Where χ_{2D} = 6.81 × 10^{-2} cm^{-1}/K is the the temperature coefficient; R = 2.0 μm is the radius of suspended graphene; $r_0 = \lambda/\pi NA =$ 0.18 μm is the radius of the laser beam spot; $t = 0.335$ nm is the thickness of graphene; α is 0.98 for the 100 × objective lens[56,57]; $\delta\omega/\delta P$ is the slope obtained from Supplementary Fig. 24d.

Consequently, the statistics of obtained thermal conductivity value of super-clean graphene and unclean graphene is presented in Supplementary Fig. 26e, which clearly confirm that the super-clean graphene exhibits higher thermal conductivity than that of unclean counterpart. Such enhancement of thermal conductivity in super-clean graphene sample can be explained by the suppression of the scattering of phonons, which could be caused by the presence of surface contamination[57], in a similar fashion as the SiO_2 substrate.

Supplementary Note 27

Discussion for Supplementary Figure 27:

We used the conventional method to transfer large-area our clean graphene samples onto 4 inch-sized SiO_2/Si wafer (Supplementary Fig. 27a). $FeCl_3$ aqueous solution was used to etch the Cu away while PMMA was used as the supporting layer. Then, the graphene film was cut into a Hall bar geometry (Supplementary Fig. 27b, inset) by large-area photolithography and oxygen plasma etching, for four-point probe measurement. 10 nm Pd and 80 nm Au were deposited on the samples using an electron-beam evaporator, followed by a standard metal lift-off technique. After the the fabrication of devices (Supplementary Fig. 27b), the sheet resistance of clean graphene is measured to be ~ 272 Ω sq^{-1}, lower than that of unclean graphene (~ 410 Ω sq^{-1}), as confirmed by statistic of the sheet resistance values (Supplementary Fig. 27c). Furthermore, the sheet resistance of large-scale continuous graphene film was characterized based on 4-inch graphene wafer (Supplementary Fig. 27d). It should be

noted that the graphene conductivity is also related to the transfer-related doping, wrinkle, and breakage, which altogether contribute to the spatial difference in sheet resistance mapping results[58].

Supplementary Table 1: Reported intensity ratio of D to G peaks in conventional Raman results.[1,15-18,59-78]

Table S1: Reported intensity ratio of D to G peaks

Group	I_D/I_G	Journal
Chan, J et al.[59]	0.25	ACS Nano
Xu, X. et al.[60]	0	Nat. Nanotechnol.
Wang, D. et al.[61]	0.06	Adv. Mater.
Kim, S. J. et al.[62]	0.025	Nano Lett.
Wang, B. et al.[63]	0.05	ACS Nano
Lee, H. C. et al.[64]	0.1	Adv. Mater.
Xiang, S. et al.[65]	0	Nano Res.
Chen, B. et al.[66]	0	Nanoscale
Li, X. et al.[67]	0.11	J. Am. Chem. Soc.
Petrone, N. et al.[68]	0	Nano Lett.
Suk, J. W. et al.[69]	0	Nano Lett.
Ma, T. et al.[70]	0.05	ACS Nano
Wu, T. et al.[71]	0	Adv. Func. Mater.
Zhou, H. et al.[18]	0	Nat. Commun.
Gao, L. et al.[72]	0	Nat. Commun.
Chen, S et al.[15]	0	Adv. Mater.
Wang, C. et al.[73]	0	Sci. Rep.
Geng, D. et al.[74]	0	Proc. Natl. Acad. Sci.
Zhang, Y. et al.[75]	0	Nano Lett.
Yan, Z. et al.[17]	0	ACS Nano
Li, X. et al.[1]	0	Science
Suk, J. W. et al.[16]	0	Nat. Nanotechnol.
Yu, Q. et al.[76]	0	Nat. Mater.
Babenko, V. et al.[77]	0	Nat. Commun.
Guo, W. et al.[78]	0	Adv. Mater.

Supplementary Table 2: Reported intensity of D to G peaks in TERS and conventional Raman results.[3,5,7,9,11,79,80]

Table S2: Reported Intensity ratio of D to G peaks		
Group	TERS	in-situ Far-field
Li, X. et al.[79]	0.8	0
Park, K. et al.[9]	0.85	0
Stadler, J. et al.[3]	6	0.1
Beams, R. et al.[7]	3.5	0
Su, W. et al.[11]	0.72	0.33
Wang, P. et al.[5]	1.3	0.5
Ghislandi, M. et al.[80]	0.5	0.6

The graphene samples were both characterized by TERS and conventional Raman spectroscopy in above works. In TERS, the D band can be caused by defect or the amorphous contamination, regardless of the preparation method (mechanical exfoliation or CVD method). Thus, the defects at the graphene domain edge, obtained by mechanical exfoliation routine can also produce a D band in TERS and no visible D peak in conventional Raman results[6,76], which also indicate the capability of TERS techniques to selectively enhance the intensity of defect-related D peak. Note that the enhancement of D band intensity in TERS varies in different works, which is dependent on TERS equipment configuration, such as the type of substrate (Au[3,5], Cu[3], glasses[6,9,11,80]), the radius and type of tips (Au[5,7,9,80] or Ag[3,6,11]), scanning probe microscopy (STM[3], AFM [5,7,9,11,80]), the excitation wavelength of laser (532 nm[5,6,11] or 632.8 nm[3,7,9]), and the polarization of laser (linear-polarized[7] or radial polarized[6,11]).

Supplementary Table 3: Reported cleanness values of graphene membranes.[16,81-88]

Table S3: Reported cleanness values of graphene membrane				
Year	Group	Journal	Cleanness ratio (%)	Figure number
2015	Azizi, A. et al.[81]	ACS Nano	42±20	1b
2013	Gong, C. et al.[82]	J. Phys. Chem. C	30±10	6
2012	Lin, Y. C. et al.[83]	Nano Lett.	10±5	2b,c
2012	O'Hern, S. C. et al.[84]	ACS Nano	35±10	6a-e
2011	Suk, J. W. et al.[16]	ACS Nano	40±5	4c
2011	Kim, K. et al.[85]	ACS Nano	20±10	2c,d
2011	Huang, P. Y. et al.[86]	Nature	30±10	2e-g, S4a
2010	Regan, W. et al.[87]	Appl. Phys. Lett	32±5	3a, 4a
2010	Alemán, B. et al.[88]	ACS Nano	50±20	S2

Note: The cleanness values were obtained from the corresponding TEM images of graphene membranes in the references given. Note that the cleanness of Ref. 84, 85, 86 is derived from STEM or dark-field TEM image of graphene. Conventionally, in TEM image, contamination indicates a darker contrast than that of clean graphene, while contamination exhibits a brighter contrast in STEM or dark-field TEM images. The cleanness of ref. 88 is derived from Supplementary Fig. 2, where the large contamination such as metal contaminants is not calculated.

Supplementary Table 4: Reported contact resistances of graphene.[38,39,89-103]

Table S4: Reported contact resistances of graphene on SiO_2/Si substrates

Group	Graphene	$R_{contact}$ (Ω μm)	Method
Venugopal, A. et al.[89]	Exfoliated G	350-3,000	TLM
Nouchi, R. et al.[90]	Exfoliated G	100-620	TLM
Nagashio, K. et al.[91]	Exfoliated G	500	4P
Kim, S. et al.[92]	Exfoliated G	500	TLM
Nagashio, K. et al.[93]	Exfoliated G	500	CBK
Blake, P. et al.[94]	Exfoliated G	250	4P
Russo, S. et al.[95]	Exfoliated G	250	TLM
Xia, F. N. et al.[96]	Exfoliated G	185	TLM
Fisichella, G. et al.[97]	CVD G	15,100	TLM
Hsu, A. et al.[98]	CVD G	200-2,500	TLM
Ha, T. J. et al.[99]	CVD G	1,900	2P
Balci, O. et al.[100]	CVD G	1,400	TLM
Franklin, A. D. et al.[101]	CVD G	715	4P
Leong, W. S. et al.[102]	CVD G	335	TLM
W. J. Yoo et al.[38]	CVD G	270	TLM
Xuelei Liang et al.[39]	CVD G	200	TLM

Note: The values of mechanically exfoliated graphene and CVD-grown graphene are summarized. The methods for measuring contact resistance are as follows: the transfer length method (TLM), four-probe method (4P), two-probe method (2P), and cross-bridge Kelvin (CBK) method.

Supplementary Table 5: Reported carrier mobilities of graphene of CVD graphene on SiO_2 substrate.[15,17,18,59-75]

Table S5: Reported Mobilities of graphene on SiO_2/Si substrates

Group	μ ($cm^2\ V^{-1}\ s^{-1}$)	Journal
Chan, J. et al.[59]	12,700	ACS Nano
Xu, X. et al.[60]	6,500	Nat. Nanotechnol.
Wang, D. et al.[61]	5,602	Adv. Mater.
Kim, S. J. et al.[62]	17,700	Nano Lett.
Wang, B. et al.[63]	10,663	ACS Nano
Lee, H. C. et al.[64]	10,335	Adv. Mater.
Xiang, S. et al.[65]	11,000	Nano Res.
Chen, B. et al.[66]	12,000	Nanoscale
Li, X. et al.[67]	4,000	J. Am. Chem. Soc.
Petrone, N. et al.[68]	25,000	Nano Lett.
Suk, J. W. et al.[69]	10,300	Nano Lett.
Ma, T. et al.[70]	13,000	ACS Nano
Wu, T. et al.[71]	8,000	Adv. Func. Mater.
Zhou, H. et al.[18]	16,000	Nat. Commun.
Gao, L. et al.[72]	7,100	Nat. Commun.
Chen, S. et al.[15]	5,200	Adv. Mater.
Wang, C. et al.[73]	2,400	Sci. Rep.
Geng, D. et al.[74]	2,500	Proc. Natl. Acad. Sci.
Zhang, Y. et al.[75]	4,200	Nano Lett.
Yan, Z. et al.[17]	11,000	ACS Nano

Supplementary Table 6: Measured carrier mobilities of graphene and unclean graphene based 12 devices.

Table S6: Measured carrier mobility ($cm^2 V^{-1} s^{-1}$)

	1	2	3	4	5	6	7	8	9	10	11	12	average
Clean G	15,900	17,600	17,500	18,000	16,900	18,600	15,800	18,000	17,900	16,500	14,900	15,800	16,950
Unclean G	10,350	10,160	14,550	9,320	16,800	9,740	9,500	1,3100	9,060	11,400	10,950	9,060	11,160

Note that the transfer-related water doping, substrate and wrinkle would all influence the carrier mobility, lead to a fluctuation in final result[32,68.] In this regard, the non-uniform spatial distribution of polymer residue would result in a large fluctuation and reduction of carrier mobility in unclean sample

Supplementary references:

1. Li, X. *et al.* Large-area synthesis of high-quality and uniform graphene films on copper foils. *Science* **324**, 1312-1314 (2009).

2. Zeng, Z. C. *et al.* Electrochemical tip-enhanced Raman spectroscopy. *J. Am. Chem. Soc.* **137**, 11928-11931 (2015).

3. Stadler, J., Schmid, T. & Zenobi, R. Nanoscale chemical imaging of single-layer graphene. *ACS Nano* **5**, 8442-8448 (2011).

4. Beams, R. *et al.* Tip-enhanced Raman mapping of local strain in graphene. *Nanotechnology* **26**, 175702 (2015).

5. Wang, P. *et al.* Reversible defect in graphene investigated by tip-enhanced Raman spectroscopy. Plasmonics 7. 555–561 (2012).

6. Su, W. & Roy, D., Visualizing graphene edges using tip-enhanced Raman spectroscopy. *Journal of Vacuum Science & Technology B, Nanotechnology and Microelectronics: Materials, Processing, Measurement, and Phenomena* **31**, 041808 (2013).

7. Beams, R., Cançado, L. G., Oh, S.-H., Jorio, A. & Novotny, L. Spatial coherence in near-field Raman scattering. *Phys. Rev. Lett.* **113.** 186101. (2014).

8. Mignuzzi, S. *et al.* Probing individual point defects in graphene via near-field Raman scattering. *Nanoscale* **7**, 19413-8 (2015).

9. Park, K. *et al.* Probing bilayer grain boundaries in large-area graphene with tip-enhanced Raman Spectroscopy. *Adv. Mater.* 29. 1603601 (2016).

10. Rickman, R. H. & Dunstan, P. R., Enhancement of lattice defect signatures in graphene andultrathin graphite using tip-enhanced Raman spectroscopy. *Journal of Raman Spectroscopy* **2014**, 45, 15-21.

11. Su, W., Kumar, N., Dai, N. & Roy, D., Nanoscale mapping of intrinsic defects in single-layer graphene using tip-enhanced Raman spectroscopy. *Chem Commun (Camb)* **2016**, 52, 8227-30.

12. Iwasaki, T. *et al.* Local hole doping concentration modulation on graphene probed by tip-enhanced Raman spectroscopy. *Carbon* **2017**, 111, 67-73.

13. Pashaee, F., Sharifi, F., Fanchini, G. & Lagugne-Labarthet, F., Tip-enhanced Raman spectroscopy of graphene-like and graphitic platelets on ultraflat gold nanoplates. *Phys. Chem. Chem. Phys.* **2015**, 17, 21315-22.

14. Zhang, R. *et al.* Optical visualization of individual ultralong carbon nanotubes by chemical vapour deposition of titanium dioxide nanoparticles. *Nat. Commun.* **4**, 1727 (2013).

15. Chen, S. *et al.* Millimeter-size single-crystal graphene by suppressing evaporative loss of Cu during low pressure chemical vapor deposition. *Adv. Mater.* **25**, 2062-2065 (2013).

16. Suk, J. W. *et al.* Transfer of CVD-grown monolayer graphene onto arbitrary substrates. *ACS Nano* **5**, 6916-6924 (2011).

17. Yan, Z. *et al.* Toward the synthesis of wafer-scale single-crystal graphene on copper foils. *ACS Nano* **6**, 9110-9117 (2012).

18. Zhou, H. *et al.* Chemical vapour deposition growth of large single crystals of monolayer and bilayer graphene. *Nat Commun.* **4**, 2096 (2013).

19. Zhang, W., Wu, P., Li, Z. & Yang, J. First-principles thermodynamics of graphene growth on Cu surfaces. *J. Phys. Chem. C* **115**, 17782-17787 (2011).

20. Yuan, Q. *et al.* Magic carbon clusters in the chemical vapor deposition growth of graphene. *J. Am. Chem. Soc.* **134**, 2970-2975 (2011).

21. Niu, T., Zhou, M., Zhang, J., Feng, Y. & Chen, W. Growth intermediates for CVD graphene on Cu (111): carbon clusters and defective graphene. *J. Am. Chem. Soc.* **135**, 8409-8414 (2013).

22. Li, X., Cai, W., Colombo, L. & Ruoff, R. S. Evolution of graphene growth on Ni and Cu by carbon isotope labeling. *Nano Lett.* **9**, 4268-4272 (2009).

23. Lin, H. C., Chen, Y. Z., Wang, Y. C. & Chueh, Y. L. The essential role of Cu vapor for the self-limit graphene via the Cu catalytic CVD method. *J. Phys. Chem. C* **119**, 6835-6842 (2015).

24. Matkovic, A. *et al.* Influence of transfer residue on the optical properties of chemical vapor deposited graphene investigated through spectroscopic ellipsometry. *J. Appl. Phys.* **114**, 093505 (2013).

25. Nair, N. N. *et al.* Fine structure constant defines visual transparency of graphene. *Science* **320**, 1308 (2008).

26. Kravets, V. G. *et al.* Spectroscopic ellipsometry of graphene and an exciton-shifted van Hove peak in absorption. *Phys. Rev. B* **81**, 155413 (2010).

27. Chae, D. *et al.* Excitonic Fano resonance in free-standing graphene. *Nano Lett.* **11**, 1379–1382 (2011).

28. Lin, W. *et al.* A Direct and Polymer-Free Method for Transferring Graphene Grown by Chemical Vapor Deposition to Any Substrate. *ACS Nano* **8**, 1784–1791 (2014).

29. Sun, Z. *et al.* Growth of graphene from solid carbon sources. *Nature* **468**, 549-552 (2010).

30. Li, X. *et al.* Transfer of large-area graphene films for high-performance transparent conductive electrodes. *Nano Lett.* **9**, 4359–4363 (2009).

31. Zhao, P. *et al*. Equilibrium chemical vapor deposition growth of Bernal-stacked bilayer graphene. *ACS Nano* **8**, 11631–11638 (2014).

32. Bae, S. *et al*. Roll-to-roll production of 30-inch graphene films for transparent electrodes. *Nat. Nanotechnol.* **5**, 574-578 (2010).

33. Gadipelli, S. *et al.* A highly practical route for large-area, single layer graphene from liquid carbon sources such as benzene and methanol. *J. Mater. Chem*. **21**, 16057-16065 (2011).

34. Kim, H. H. *et al*. Clean transfer of wafer-scale graphene via liquid phase removal of polycyclic aromatic hydrocarbons. *ACS Nano* **9**, 4726–4733 (2015).

35. Fang, W. *et al*. Asymmetric growth of bilayer graphene on copper enclosures using low pressure chemical vapor deposition. *ACS Nano* **8**, 6491–6499 (2014).

36. Kim, K. *et al.* Selective metal deposition at graphene line defects by atomic layer deposition. *Nat. Commun.* **5**, 4781 (2014).

37. Yu, S. U. *et al.* Simultaneous visualization of graphene grain boundaries and wrinkles with structural information by gold deposition. *ACS Nano* **8**, 8662-8668 (2014).

38. Yue, D., Ra, C., Liu, X., Lee, D. & Yoo, W. Edge contacts of graphene formed by using a controlled plasma treatment. *Nanoscale* **7**, 825-831 (2015).

39. Li, W. et al. Ultraviolet/ozone treatment to reduce metal-graphene contact resistance. *Appl. Phys. Lett.* **102**, 183110 (2013).

40. Jia, Y. *et al.* Toward high carrier mobility and low contact resistance: laser cleaning of PMMA residues on graphene surfaces. *Nano-Micro Lett.* **8**, 336–346 (2016).

41. Gahng, S. *et al.* Reduction of metal contact resistance of graphene devices via CO_2 cluster cleaning. *Appl. Phys. Lett.* **104,** 223110 (2014).

42. Li, W. *et al.* Highly reproducible and reliable metal/graphene contact by ultraviolet-ozone treatment. *J. appl. Phys.* **115,** 114304 (2014).

43. Chen, J., Jang, C., Xiao, S., Ishigami, M. & Fuhrer, M. Intrinsic and extrinsic performance limits of graphene devices on SiO_2. *Nat. Nanotechnol*. **3**, 206-209 (2008).

44. Martin, J. *et al.* Observation of electron–hole puddles in graphene using a scanning single-electron transistor. *Nat. Phys*. **4**, 144-148 (2008).

45. Banszerus, L. *et al.* Ultrahigh-mobility graphene devices from chemical vapor deposition on reusable copper. *Sci. Adv.* **1**, e1500222 (2015).

46. Banszerus, L. *et al.* Ballistic transport exceeding 28 μm in CVD grown graphene. *Nano Lett.* **16**, 1387-1391 (2016).

47. Mayorov, A. S. *et al.* Micrometer-scale ballistic transport in encapsulated graphene at Room temperature. *Nano Lett.* **11**, 2396-2399 (2011).

48. Wang, L. *et al.* One-dimensional electrical contact to a two-dimensional material. *Science* **342**, 614-617 (2013).

49. Dean, C. R. *et al.* Boron nitride substrates for high-quality graphene electronics. *Nature Nanotechnol.* **5**, 722-726 (2010).

50. Haigh, S. J. *et al.* Cross-sectional imaging of individual layers and buried interfaces of graphene-based heterostructures and superlattices. *Nat. Mater.* **11**, 764-767 (2012).

51. Schwierz, F. Graphene transistors. *Nat. Nanotechnol.* **5**, 487-496 (2010).

52. Robinson, J. A. *et al.* Correlating raman spectral signatures with carrier mobility in epitaxial graphene: a guide to achieving high mobility on the wafer scale. *Nano Lett.* **9**, 2873-2876 (2009).

53. Neumann, C. *et al.* Raman spectroscopy as probe of nanometre-scale strain variations in graphene. *Nat. Commun.* **6**, 9429 (2015).

54. Lee, J. E., Ahn, G., Shim, J., Lee, Y. S. & Ryu, S. Optical separation of mechanical strain from charge doping in graphene. *Nat. Commun.* **3**, 1024 (2012).

55 Li, Z. *et al.* Effect of airborne contaminants on the wettability of supported graphene and graphite. *Nat. Mater*. **12**, 925-931 (2013).

56. Cai, W. *et al.* Thermal transport in suspended and supported monolayer graphene grown by chemical vapor deposition. *Nano Lett* **10**, 1645-1651 (2010).

57. Pettes, M. T., Jo, I., Yao, Z. & Shi, L. Influence of polymeric residue on the thermal conductivity of suspended bilayer graphene. *Nano Lett* **11**, 1195-1200 (2011).

58. Petrone, M. *et al.* Chemical vapor deposition-derived graphene with electrical performance of exfoliated graphene. *Nano Lett.* **12**, 2751–2756 (2012).

59. Chan, J. *et al.* Reducing extrinsic performance limiting factors in graphene grown by chemical vapor deposition. *ACS Nano* **4**, 3224-3229 (2012).

60. Xu, X. *et al.* Ultrafast growth of single-crystal graphene assisted by a continuous oxygen supply. *Nat. Nanotechnol.* **132**, 1-6 (2016).

61. Wang, D. *et al.* Clean-lifting transfer of large-area residual-free graphene films. *Adv. Mater.* **25,** 4521–4526 (2013),

62. Kim, S. J. *et al.* Ultraclean patterned transfer of single-layer graphene by recyclable pressure sensitive adhesive films. *Nano lett.* **15**, 32336-3240 (2015).

63. Wang, B. *et al.* Support-free transfer of ultrasmooth graphene films facilitated by self-assembled monolayers for electronic devices and patterns. *ACS Nano* **10**. 1404-1410 (2016).

64. Lee, H. C. *et al.* Facet-mediated growth of high-quality monolayer graphene on arbitrarily rough copper surfaces. *Adv. Mater.* **28**, 2010-2017 (2016).

65. Xiang, S. *et al*, Low-temperature quantum transport in CVD-grown single crystal graphene. *Nano Res.* **9**, 1823-1830 (2016).

66. Chen, B. *et al.* How good can CVD-grown monolayer graphene be? *Nanoscale* **6**, 15255-15261 (2014).

67. Li, X. *et al.* Large-area graphene single crystals grown by low-pressure chemical vapor deposition of methane on copper. *J. Am. Chem. Soc.* **133**, 2816-9 (2011).

68. Petrone, N. *et al.* Chemical vapor deposition-derived graphene with electrical performance of exfoliated graphene. *Nano lett.* **12**, 2751-2756 (2012).

69. Suk, J. W. *et al.* Enhancement of the electrical properties of graphene grown by chemical vapor deposition via controlling the effects of polymer residue. *Nano Lett.* **13**, 1462-1467 (2013).

70. Ma, T. *et al.* Repeated growth-etching-regrowth for large-area defect-free single crystal graphene by chemical vapor deposition. *ACS Nano* **12**, 12806-12813 (2014).

71. Wu, T. *et al.* Triggering the continuous growth of graphene toward millimeter-sized grains. *Adv. Funct. Mater.* **23**, 198-203 (2013).

72. Gao, L. *et al.* Repeated growth and bubbling transfer of graphene with millimetre-size single-crystal grains using platinum. *Nat. Commun.* **3,** 699 (2012).

73. Wang, C. *et al.* Growth of millimeter-size single crystal graphene on Cu foils by circumfluence chemical vapor deposition. *Sci. Rep.* **4**, 4537 (2014).

74. Geng, D. *et al.* Uniform hexagonal graphene flakes and films grown on liquid copper surface. *Proc. Natl. Acad. Sci.* **109**, 7992-7996 (2012).

75. Zhang, Y. *et al.* Vapor trapping growth of single-crystalline graphene flowers: synthesis, morphology, and electronic Properties. *Nano Lett*. **12**. 2810-2816 (2010).

76. Yu, Q. *et al.* Control and characterization of individual grains and grain boundaries in graphene grown by chemical vapour deposition. *Nat. Mater.* **10**. 443-449 (2011).

77. Babenko, V. *et al.* Rapid epitaxy-free graphene synthesis onsilicidated polycrystalline platinum. *Nat. commun.* **6**. 7536 (2015).

78. Guo, W. *et al.* Oxidative-etching-assisted synthesis of centimeter-Sized single-crystalline graphene. *Adv. Mater.* **28**. 3152-3158 (2016).

79. Li, X. *et al.* Investigation on tip enhanced Raman spectra of graphene. *Spectrochimica Acta Part A: Molecular and Biomolecular Spectroscopy*, **190.** 378-382 (2017).

80. Ghislandi, M., Hoffmann, G. G., Tkalya, E., Xue, L. & With, D. G. Tip-enhanced Raman spectroscopy and mapping of graphene sheets. *Appl. Spec.Rev.* **47.** 371–381 (2012).

81. Azizi, A. *et al.* Freestanding van der Waals heterostructures of graphene and transition metal dichalcogenides. *ACS Nano* **9**, 4882-4890 (2015).

82. Gong, C. *et al.* Rapid selective etching of PMMA residues from transferred graphene by carbon dioxide. *The J. Phys. Chem. C* **117**, 23000-23008 (2013).

83. Lin, Y. C. *et al.* Graphene annealing: how clean can it be? *Nano Lett*. **12**, 414-419 (2012).

84. O'Hern, S. C. *et al.* Selective molecular transport through intrinsic defects in a single layer of CVD graphene. *ACS Nano* **6**, 10130-10138 (2012).

85. Kim, K. *et al.* Grain boundary mapping in polycrystalline graphene. *ACS Nano* **5**, 2142-2146 (2011).

86. Huang, P. Y. *et al.* Grains and grain boundaries in single-layer graphene atomic patchwork quilts. *Nature* **469**, 389-392 (2011).

87. Regan, W. *et al.* A direct transfer of layer-area graphene. *Appl. Phys. Lett.* **96**, 113102 (2010).

88. Alemán, B. *et al.* Transfer-free batch fabrication of large-area suspended graphene membranes. *ACS Nano* **4**, 4762-4768 (2010).

89. Venugopal, A., Colombo, L. & Vogel, E. Contact resistance in few and multilayer graphene devices. *Appl. Phys. Lett.* **96**, 013512 (2010).

90. Nouchi, R., Saito, T. & Tanigaki, K. Observation of negative contact resistances in graphene field-effect transistors. *J. Appl. Phys*. **111**, 084314 (2012).

91. Nagashio, K., Nishimura, T., K. Kita, & Toriumi, A. "Metal/graphene contact as a performance Killer of ultra-high mobility graphene analysis of intrinsic mobility and contact resistance," in *Proc. IEEE Int. Electron Devices Meeting (IEDM)*, Dec. 2009, pp. 1-4.

92. Kim, S. *et al.* Realization of a high mobility dual-gated graphene field-effect transistor with Al_2O_3 dielectric. *Appl. Phys. Lett.* **94**, 062107 (2009).

93. Nagashio, K., Nishimura, T., Kita, K. & Toriumi, A. Contact resistivity and current flow path at metal/graphene contact. *Appl. Phys. Lett.* **97**, 143514 (2010).

94. Blake, P. *et al.* Influence of metal contacts and charge inhomogeneity on transport properties of graphene near the neutrality point. *Solid State Commun.* **149**, 1068-1071 (2009).

95. Russo, S., Craciun, M., Yamamoto, M., Morpurgo, A. & Tarucha, S. Contact resistance in graphene-based devices. *Physica E* **42**, 677-679 (2010).

96. Xia, F. N., Perebeinos, V., Lin, Y. M., Wu, Y. Q. & Avouris, P. The origins and limits of metal-graphene junction resistance. *Nat. Nanotechnol.* **6**, 179-184 (2011).

97. Fisichella, G. *et al.* Micro-and nanoscale electrical characterization of large-area graphene transferred to functional substrates. *Beilstein J. Nanotechnol.* **4**, 234-242 (2013).

98. Hsu, A., Wang, H., Kim, K. K., Kong, J. & Palacios, T. Impact of graphene interface quality on contact resistance and RF device performance. *IEEE Electron Device Lett.* **2**, 1008-1010 (2011).

99. Ha, T. J., Akinwande, D. & Dodabalapur, A. Hybrid graphene/organic semiconductor field-effect transistors. *Appl. Phys. Lett.* **101**, 033309 (2012).

100. Balci, O. & Kocabas,C. Rapid thermal annealing of graphene-metal contact. *Appl Phys Lett* **101**, 243105 (2012).

101. Franklin, A. D., Han, S. J., Bol, A. A. & Perebeinos, V. Double contacts for improved performance of graphene transistors. *IEEE Electron Device Lett.* **33**, 17-19 (2012).

102. Leong, W. S., Nai, C. T. & J. T. Thong, What does annealing do to metal–graphene contacts? *Nano Lett.* **14**, 3840-3847 (2014).
