# Supplementary material for: Towards super-clean graphene
Source: Nat Commun. 2019 Apr 23;10:1912. doi: 10.1038/s41467-019-09565-4 (PMC6478734; doi:10.1038/s41467-019-09565-4)
Supplement: Supplementary file 2 — Peer Review File [file 41467_2019_9565_MOESM2_ESM.pdf]

Editorial Note: This manuscript has been previously reviewed at another journal that is not operating a transparent peer review scheme. This document only contains reviewer comments and rebuttal letters for versions considered at *Nature Communications*.
